# Optimizing Weight Loss in the GLP-1 Era: Preserving Muscle Mass, Function and Metabolic Health Through Precision Nutrition and Resistance Training

**DOI:** 10.3390/ph19060897

**Published:** 2026-06-05

**Authors:** Edgar Sancho-Haro, Mario Muñoz-López, Eneko Baz-Valle, Carlota Valeria Villanueva-Tobaldo, José Francisco Tornero-Aguilera, José Francisco López-Gil, Miguel López-Moreno, Alexandra Martín-Rodríguez, Vicente Javier Clemente-Suárez

**Affiliations:** 1 Department of Sport Sciences, Faculty of Sport and Health Sciences, Fit Generation Research Institute, AD400 Andorra la Vella, Andorra; edgar.sancho@fitgeneration.es (E.S.-H.); jtornero@fitgeneration.es (J.F.T.-A.); 2 Department of Nutrition and Dietetics, Faculty of Sport and Health Sciences, Fit Generation Research Institute, AD400 Andorra la Vella, Andorra; 3 School of Medicine, Universidad Espíritu Santo, Samborondón 09-01-952, Ecuador; josefranciscolopezgil@gmail.com; 4 Faculty of Health Sciences, Universidad Autónoma de Chile, Temuco 4780000, Chile; 5 Diet, Planetary Health and Performance, Faculty of Health Sciences, Universidad Francisco de Vitoria, 28223 Madrid, Spain; 6 Faculty of Education Sciences, UNIE University, 28015 Madrid, Spain; 7 Grupo de Investigación en Cultura, Educación y Sociedad, Universidad de la Costa, Barranquilla 080002, Colombia

**Keywords:** GLP-1 receptor agonists, high-quality weight loss, skeletal muscle preservation, precision protein nutrition, resistance training

## Abstract

The emergence of glucagon-like peptide-1 receptor agonists (GLP-1RAs) and dual incretin-based therapies has fundamentally transformed obesity pharmacotherapy, enabling magnitudes of non-surgical weight loss that were previously unattainable. Yet, the clinical success of these treatments cannot be measured in kilograms alone. Total body weight is a composite, tissue-nonspecific endpoint that fails to distinguish between adipose reduction and losses in skeletal muscle mass, strength, and physical function—compartments of direct relevance to metabolic health, functional independence, and long-term resilience. This narrative review builds on and extends existing conceptualizations of weight loss quality by proposing a clinically oriented, multidimensional framework of high-quality weight loss. Within this framework, preferential adiposity reduction is achieved while preserving skeletal muscle mass, neuromuscular function, dietary adequacy, and cardiometabolic health. We examine the physiological and clinical consequences of lean tissue loss during pharmacological energy restriction, with specific attention to phenotypes at greatest risk (i.e., older adults, individuals with sarcopenic obesity, and those with type 2 diabetes). We then evaluate the evidence supporting precision protein nutrition, dietary fiber adequacy, and gastrointestinal tolerability management as nutritional countermeasures, followed by a mechanistic and clinical analysis of resistance training as the primary exercise strategy for preserving lean mass and function. Finally, we discuss body composition monitoring, integrated multidisciplinary care, and unresolved research gaps. The future of obesity treatment lies not in greater weight loss per se, but in achieving better weight loss—defined as metabolically favorable, functionally responsible, and clinically sustainable. Bone health is treated as a further dimension of high-quality weight loss, since pharmacologically driven energy restriction can adversely affect areal bone mineral density and microarchitecture, and adequate protein intake combined with mechanical loading is required to preserve skeletal integrity alongside lean mass.

## 1. Introduction: The GLP-1 Era and the Redefinition of Weight Loss

Obesity is now widely recognized as a chronic, relapsing, and progressive disease with consequences that extend well beyond excess body mass itself, affecting metabolic, cardiovascular, mechanical, and psychosocial health [1]. This shift in understanding has important therapeutic consequences. Obesity management can no longer be framed simply as a short-term effort to reduce body weight; rather, it requires a long-term strategy aimed at improving health, reducing disease burden, and preserving functional capacity over time [1]. Yet for many years, the practical treatment of obesity remained constrained by the limited efficacy of lifestyle intervention alone in a substantial proportion of patients and by the modest effectiveness or tolerability of earlier pharmacological options.

That therapeutic landscape has changed markedly with the emergence of glucagon-like peptide-1 receptor agonists (GLP-1RAs) and related incretin-based therapies. These agents have redefined expectations for non-surgical obesity treatment by producing magnitudes of weight loss that were previously difficult to achieve without bariatric procedures [2,3]. In adults with overweight or obesity, semaglutide 2.4 mg has been shown to induce substantial and clinically meaningful weight reduction [2], while tirzepatide, a dual glucose-dependent insulinotropic polypeptide (GIP) and glucagon-like peptide-1 (GLP-1) receptor agonist, has yielded even greater body weight reductions in phase 3 obesity trials [3]. The significance of this pharmacological shift extends beyond the scale. In people with overweight or obesity and established cardiovascular disease, semaglutide has also demonstrated cardiovascular benefit, underscoring that effective obesity treatment carries implications that are broader than body mass reduction alone [4].

The term GLP-1 era refers here to the current clinical period defined by the widespread availability and high efficacy of incretin-based anti-obesity pharmacotherapies. It encompasses both selective GLP-1 receptor agonists (GLP-1RAs), of which liraglutide and semaglutide are the most prominent representatives, and dual-incretin agonists such as tirzepatide, which co-activates the GLP-1 and glucose-dependent insulinotropic polypeptide (GIP) receptors. The two drug classes converge on appetite suppression, delayed gastric emptying, and reduced energy intake, but they differ in important mechanistic and clinical respects. Dual GLP-1/GIP agonism typically produces a larger absolute weight loss and a more pronounced absolute reduction in fat-free mass than selective GLP-1RA monotherapy, in line with the broader impact of GIP co-agonism on adipocyte metabolism and overall energy balance. This distinction is clinically consequential: the larger the absolute weight loss, the greater the absolute amount of lean tissue at risk during energy restriction, and the more important the nutritional and exercise countermeasures discussed in the sections that follow.

As a result, the central question in obesity care is changing. The field is no longer asking only whether substantial non-surgical weight loss is possible; it is increasingly asking whether the quality of that weight loss is biologically and clinically favorable [2,3,4,5]. This distinction is crucial. Percentage body weight reduction remains the most visible and widely used endpoint in both clinical trials and routine practice [1,2], but body weight is a composite measure. It does not distinguish between reductions in adipose tissue and losses in non-fat compartments, nor does it indicate whether the observed change reflects a favorable remodeling of body composition or a more problematic erosion of metabolically and functionally relevant tissue [5]. In the era of potent incretin-based pharmacotherapy, this limitation becomes more, not less, important, precisely because treatment-induced change is now large enough to alter body composition in clinically meaningful ways [5].

This issue becomes even more relevant when skeletal muscle is considered. Skeletal muscle is not simply a locomotor tissue. It plays a central role in glucose disposal, insulin sensitivity, resting energy expenditure, and overall metabolic resilience, particularly during aging and chronic disease [6]. Its clinical importance is reflected in current sarcopenia frameworks. The revised European Working Group on Sarcopenia in Older People (EWGSOP2) identifies low muscle strength as the key feature of probable sarcopenia, uses muscle quantity and quality to confirm the diagnosis, and considers reduced physical performance an indicator of greater severity [7]. Likewise, the European Society for Clinical Nutrition and Metabolism (ESPEN) and European Association for the Study of Obesity (EASO) consensus on sarcopenic obesity emphasizes that excess adiposity may coexist with reduced muscle mass and impaired muscle function, creating a phenotype with distinct metabolic and functional consequences [8]. These frameworks are directly relevant to obesity treatment because they make clear that therapeutic success cannot be judged only by the amount of weight lost, but must also be judged by whether the tissues and functions most relevant to long-term health are being preserved.

The concern is not that every reduction in fat-free mass during weight loss should be viewed as pathological. Some decline in non-fat compartments is expected during negative energy balance, and fat-free mass itself is not synonymous with skeletal muscle mass [5,6]. Nevertheless, this is not a trivial issue. In individuals with lower baseline muscle reserve, older age, type 2 diabetes, sarcopenic obesity, sedentary behavior, or already impaired physical function, even modest losses in non-fat tissue may have greater implications for strength, mobility, and independence than in younger individuals with more robust reserve [6,7,8]. The biological meaning of weight loss therefore depends not only on how much mass is lost, but also on which tissue is lost, in what clinical context, and with what functional consequence.

The GLP-1RA era has sharpened this concern because the same mechanisms that make these therapies effective may also introduce nutritional and behavioral vulnerabilities if treatment is not accompanied by structured support [9,10]. By reducing appetite, enhancing satiety, and delaying gastric emptying, GLP-1RAs lower energy intake and facilitate weight loss [2,3,9]. Yet those same effects may also reduce overall food intake to the point that dietary adequacy becomes suboptimal, especially when appetite suppression, meal compression, or gastrointestinal symptoms are not actively managed [9,10]. In practice, pharmacological efficacy does not automatically guarantee nutritional quality. A patient may lose weight successfully while progressively drifting toward low protein intake, lower dietary fiber exposure, reduced meal structure, and diminished capacity to sustain resistance exercise unless these issues are addressed intentionally [9,10].

For that reason, the concept of high-quality weight loss is becoming increasingly important. In broad terms, high-quality weight loss refers to a pattern of treatment response in which excess and metabolically harmful adiposity is reduced while skeletal muscle-related reserve, muscle quality, strength, physical function, and nutritional adequacy are preserved as much as possible [5,6,7,8]. This perspective is consistent with evidence showing that higher protein intake during weight loss can attenuate declines in muscle mass in adults with overweight or obesity [11], and that resistance training provides the most direct non-pharmacological stimulus for preserving lean tissue and neuromuscular function during caloric restriction [12]. However, these intervention strategies only make full sense once the field accepts a more fundamental point: successful obesity treatment can no longer be defined by total body weight reduction alone.

Accordingly, the aim of this narrative review is to critically examine how GLP-1-based anti-obesity pharmacotherapy is reshaping the meaning of successful weight loss, with particular emphasis on the preservation of lean mass, skeletal muscle quality, strength, physical function, and metabolic health through precision nutrition and resistance training [2,5,9]. Specifically, this review discusses why body weight alone is no longer an adequate endpoint; examines the physiological and clinical significance of lean mass loss during pharmacological energy restriction; develops the concept of high-quality weight loss as a new clinical and performance-oriented framework; evaluates the role of protein nutrition, dietary fiber, and gastrointestinal tolerance in supporting dietary adequacy and adherence; examines resistance training as the main countermeasure to disproportionate muscle-related decline; and highlights the importance of monitoring body composition and function rather than body weight alone [5,6,7,8,9,10,11,12]. In doing so, the review argues that the future of obesity care lies not simply in achieving greater weight loss, but in achieving better weight loss—weight loss that is metabolically favorable, functionally responsible, and clinically sustainable.

## 2. Methods

This manuscript was developed as a narrative review designed to critically examine how GLP-1RAs and related anti-obesity pharmacotherapies are reshaping the concept of successful weight loss, with particular attention to body composition, skeletal muscle preservation, physical function, precision nutrition, dietary fiber, gastrointestinal tolerance, resistance training, and multidimensional outcome monitoring [2,3,5,9,10]. Rather than following the structure of a formal systematic review, the objective was to build a conceptually integrated and clinically relevant synthesis of the literature that could support a mechanistically grounded discussion of high-quality weight loss in the current therapeutic era.

The literature search was conducted using PubMed/MEDLINE, Web of Science, and Scopus. Additional articles were identified through manual screening of the reference lists of key original investigations, narrative reviews, systematic reviews, meta-analyses, consensus statements, and position papers when these were considered central to the conceptual development of the manuscript [5,7,8,9,10]. Search terms were combined using Boolean operators and included, among others, the following expressions: “GLP-1 receptor agonist”, “semaglutide”, “liraglutide”, “tirzepatide”, “obesity”, “weight loss”, “body composition”, “fat mass”, “fat-free mass”, “lean mass”, “skeletal muscle”, “appendicular lean mass”, “muscle strength”, “physical performance”, “sarcopenia”, “sarcopenic obesity”, “dietary protein”, “protein intake”, “anabolic resistance”, “dietary fiber”, “satiety”, “gastrointestinal tolerance”, “resistance training”, and “exercise intervention” [5,7,8,9,10,11,12].

No rigid lower time limit was imposed on the search. However, greater emphasis was placed on recent literature for GLP-1-based pharmacotherapy, supportive care recommendations, and contemporary obesity treatment paradigms, while earlier landmark papers were retained when they were necessary to frame foundational concepts such as sarcopenia, anabolic resistance, body-composition assessment, or resistance-training physiology [5,6,7,8,9,10,11,12]. Priority was given to randomized controlled trials, controlled clinical studies, prospective human studies, systematic reviews, meta-analyses, consensus statements, and position papers relevant to obesity treatment, clinical nutrition, body composition, muscle physiology, sarcopenia, and exercise science [2,3,4,5,6,7,8,9,10,11,12]. Mechanistic animal and in vitro studies were considered when useful to clarify biological pathways relevant to muscle preservation, energy restriction, satiety regulation, gastrointestinal tolerance, and functional decline, but human evidence was prioritized whenever available [5,6,9,10,11,12].

The final selection of studies was guided by their direct relevance to the scientific aims of the review. Articles were included when they contributed meaningfully to one or more of the following domains: GLP-1-based obesity treatment; body composition and lean mass changes during intentional weight loss; skeletal muscle, strength, or physical performance outcomes; dietary protein and anabolic support during energy restriction; dietary fiber, satiety, and gastrointestinal tolerability; resistance training as a countermeasure to muscle-related decline; and monitoring strategies beyond total body weight [5,6,7,8,9,10,11,12]. Publications were excluded when they were clearly unrelated to obesity treatment, nutritional management, body composition, skeletal muscle, or exercise-related outcomes, or when they lacked clear relevance to the mechanistic or translational scope of the review [5,9,10]. The literature was then organized according to the conceptual structure of the manuscript so that each section could contribute to a unified scientific argument rather than to a collection of isolated summaries [5,9,10].

The literature search spanned January 2000 to October 2026, with particular weight placed on publications from 2018 onwards because these capture the bulk of the high-quality GLP-1RA-specific evidence. Titles and abstracts were screened independently by two authors, with disagreements resolved by discussion before proceeding to full-text review. Articles were retained when they were peer-reviewed primary studies, systematic reviews, meta-analyses, or consensus statements that addressed at least one of the conceptual domains examined here, when they reported human data, or when they offered mechanistic preclinical insight indispensable for biology not yet resolvable in humans. Non-peer-reviewed material, work lacking body-composition, muscle, dietary, or functional outcomes, and duplicate datasets were excluded. In integrating findings across this corpus, the greatest weight was given to meta-analyses and large randomised trials, with progressively less weight given to smaller trials, prospective controlled cohorts, observational or retrospective studies, and finally to mechanistic preclinical work. The strength of the evidence supporting each major claim is stated explicitly throughout the text, and conclusions resting on indirect or extrapolated data are flagged accordingly.

## 3. Why Body Weight Alone Is No Longer Enough

For decades, the clinical evaluation of obesity treatment has relied heavily on total body weight. This is understandable. Body weight is inexpensive to obtain, easy to reproduce, and familiar to clinicians, researchers, and patients alike [1,2]. It also carries undeniable clinical relevance: in many individuals with excess adiposity, reductions in body weight are accompanied by improvements in glycemic control, blood pressure, inflammatory burden, and mechanical stress on weight-bearing structures [1,2,3,4]. Yet the convenience of body weight has always concealed an important limitation. Body weight is a composite measure, not a tissue-specific outcome. It offers no direct insight into whether treatment is primarily reducing fat mass, depleting non-fat compartments, altering body water, or changing several compartments at once [5]. In the era of highly effective GLP-1RA-based therapies, this limitation has become much harder to ignore, because the magnitude of pharmacologically induced weight loss is now large enough to produce clinically meaningful changes in body composition [2,3,5].

**Figure 1:**
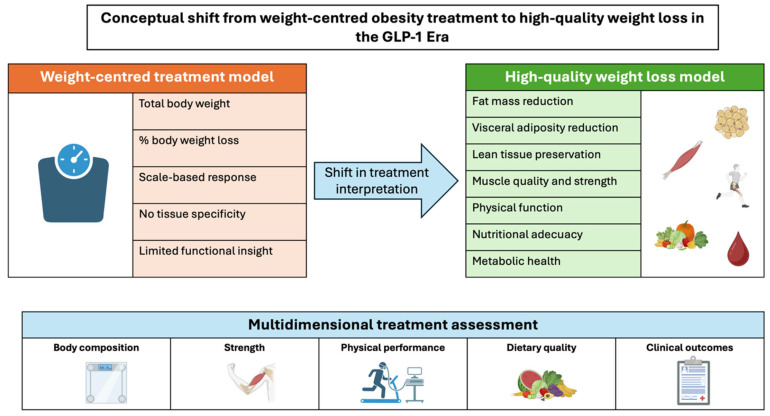
Conceptual shift from weight-centric evaluation to high-quality weight-loss assessment in the glucagon-like peptide-1 (GLP-1) era. Total body weight remains a useful clinical marker, but it is biologically non-specific because it does not distinguish between fat mass, fat-free mass, body water, and bone mass. In the setting of GLP-1RA-based obesity treatment, treatment success should be interpreted using a multidimensional framework that includes adiposity-related outcomes, muscle-related outcomes, and nutritional and clinical context. This model better reflects the biological quality of weight loss and the preservation of metabolically and functionally relevant tissues.

As obesity treatment becomes more effective, the question can no longer be limited to whether body weight decreases. The more relevant question is what kind of tissue is being lost and whether that tissue change is biologically favorable [5,6]. That distinction matters because the therapeutic goal of obesity management is not indiscriminate mass reduction, but selective improvement in body composition and long-term health. From that perspective, loss of visceral and ectopic adiposity is clearly desirable, given its close association with insulin resistance, systemic inflammation, cardiometabolic risk, and organ dysfunction [2,3,4,5,6]. By contrast, disproportionate loss of lean tissue may compromise physical reserve, reduce metabolic resilience, and increase vulnerability in patients who are already close to the threshold of functional decline [6,7,8]. For this reason, body weight alone is no longer an adequate endpoint for defining therapeutic success. The conceptual shift from a weight-centric model to a body composition- and function-oriented interpretation of obesity treatment is summarized in Figure 1.

### 3.1. Body Weight as a Crude but Incomplete Clinical Endpoint

Body weight still has an important place in obesity care. It captures the overall response to treatment, tracks broad changes in energy balance, and remains the primary outcome in most pharmacotherapy trials [1,2,3,4]. In practical terms, it has also helped define the therapeutic relevance of current anti-obesity medications. The large reductions in body weight reported with semaglutide and tirzepatide would have been difficult to achieve with earlier non-surgical treatments and have therefore changed expectations in the field [2,3]. However, the visibility of body weight as an outcome should not be mistaken for biological precision. A 10% or 15% reduction in body weight does not tell us whether the response is being driven mainly by fat mass reduction, by a mixture of fat and non-fat tissue loss, or by shifts in hydration and glycogen-associated mass [5].

This matters because similar changes on the scale can reflect very different biological responses. Two patients may lose the same percentage of body weight and yet experience very different clinical consequences depending on the composition of that loss [5,6]. One patient may lose predominantly fat mass, especially from central or ectopic depots, and therefore achieve a clearly favorable improvement in metabolic health while preserving physical capacity. Another patient may lose the same amount of total body weight but with a larger relative decline in non-fat tissue, making the response less favorable, particularly if muscle reserve was already limited at baseline [6,7,8]. In that setting, the scale records an apparently equivalent success while concealing important differences in tissue partitioning and functional consequence.

A further limitation is that body weight says nothing about regional tissue distribution. This is not a minor detail. Not all adipose tissue carries the same metabolic significance, and not all lean tissue contributes equally to function [5]. Visceral adiposity is more strongly associated with insulin resistance, dyslipidemia, hepatic steatosis, and cardiometabolic disease than total body weight alone, whereas appendicular lean mass and related muscle distribution are more closely linked to mobility, physical independence, and tolerance to activity [5,7,8]. As a result, a marked reduction in body weight may still leave unanswered the clinically important question of whether the most pathogenic fat depots are being reduced and whether the most functionally relevant lean compartments are being preserved.

This is precisely why a weight-centric model becomes less informative as treatment becomes more potent. When interventions produced only modest reductions in body weight, the limitations of the scale were easier to overlook. In the current therapeutic context, where pharmacological treatment can induce large changes in energy balance and body composition, those limitations become clinically consequential [2,3,5]. Total body weight remains a useful marker, but it should now be regarded as an initial descriptor of response rather than a complete account of treatment quality. The distinction between the quantity and the quality of weight loss is therefore no longer a conceptual refinement; it now sits at the center of how contemporary obesity treatment should be interpreted.

### 3.2. What Body Weight Conceals: Fat Mass, Fat-Free Mass, and Skeletal Muscle

One reason body weight remains such an incomplete endpoint is that the compartments underlying weight loss are often discussed imprecisely. Terms such as *lean mass*, *fat-free mass*, and *skeletal muscle mass* are sometimes used interchangeably in both clinical discussion and parts of the scientific literature, even though they refer to different physiological entities [5]. Fat-free mass includes all non-fat components of the body, including skeletal muscle, body water, connective tissue, and internal organs. Likewise, lean soft tissue measured by DXA is not equivalent to direct measurement of contractile skeletal muscle [5]. This distinction is fundamental because a decline in fat-free mass during weight loss does not necessarily mean that a proportional amount of physiologically meaningful skeletal muscle has been lost.

This is more than a semantic issue. It shapes how pharmacological weight loss is interpreted. During energy restriction, some reduction in fat-free mass is expected even under relatively favorable conditions, partly because adipose tissue itself contains a fat-free component and partly because shifts in glycogen and hydration contribute measurably to non-fat compartments [5]. For that reason, the mere presence of lean tissue decline should not automatically be treated as evidence of treatment failure or pathological muscle wasting. The more relevant question is whether tissue loss becomes disproportionate, whether functionally important compartments are being affected, and whether these changes are occurring in a patient for whom reserve is already limited [5,6,7,8].

This issue becomes especially relevant in the GLP-1RA era. Data from obesity pharmacotherapy trials and body-composition substudies consistently show that incretin-based therapies reduce both fat mass and fat-free mass, but that the predominant share of total weight loss is generally attributable to adipose tissue [2,3,5]. That point is important because public discussion has often drifted toward simplistic extremes. GLP-1-based treatment has sometimes been portrayed as producing either “pure fat loss” or, conversely, “dangerous muscle loss.” Neither interpretation is supported when body composition is examined carefully [5]. A more accurate reading of the evidence is that adiposity is reduced substantially, non-fat compartments also change to some extent, and the biological meaning of those changes depends on context rather than on the number alone [5,6].

This is why body-composition assessment is so valuable. Techniques such as DXA, bioelectrical impedance analysis, magnetic resonance imaging, and computed tomography do not provide identical information, but all are more informative than body weight alone when the aim is to understand what tissue is actually being lost [5]. Even then, interpretation should not stop at total lean mass. Regional measures such as appendicular lean mass may offer better functional insight, while indices of muscle quality and intramuscular fat can add information that tissue quantity alone cannot provide [5,7,8]. Once treatment becomes powerful enough to reshape body composition substantially, the scale stops being a sufficient proxy for what is biologically happening. Better treatment therefore requires better phenotyping.

### 3.3. Clinical Consequences of a Weight-Centric Model

The limitations of body weight become even more apparent when clinical heterogeneity is taken seriously. The same degree of non-fat tissue loss is unlikely to have the same significance in all patients [6,7,8]. In younger individuals with marked adiposity, preserved muscle reserve, and good functional capacity, modest reductions in fat-free mass may reflect an expected adaptation to reduced body size and lower mechanical loading [5,6]. In contrast, in older adults, sedentary individuals, patients with type 2 diabetes, or those with sarcopenic obesity, similar changes may occur in the setting of lower reserve, impaired muscle quality, and already compromised function [6,7,8]. In such cases, a purely weight-based interpretation can obscure clinically relevant vulnerability.

This is one reason why contemporary sarcopenia frameworks have moved away from relying on muscle quantity alone. EWGSOP2 identifies muscle strength as the principal indicator of probable sarcopenia, using muscle quantity and quality to confirm the diagnosis and physical performance to indicate severity [7]. The ESPEN/EASO consensus on sarcopenic obesity likewise emphasizes the coexistence of excess adiposity and muscle dysfunction as a clinically important phenotype that cannot be identified through body weight alone [8]. These frameworks matter here because they show, very clearly, that total body weight tells us little about whether a patient is preserving the very functions that underpin mobility, independence, and resilience.

A weight-centric model can therefore misclassify both success and risk. A patient who loses a large amount of body weight may be described as an excellent responder even if the response is biologically incomplete or functionally suboptimal. Conversely, a patient with more modest total weight loss may still achieve a highly favorable outcome if disease-driving adiposity is reduced substantially, metabolic health improves, and physical function is maintained [5,6,7,8]. The implication is straightforward: the biological success of obesity treatment cannot be inferred from the scale alone. It requires interpretation through body composition, functional status, and clinical context.

For these reasons, the evaluation of obesity treatment must move toward a multidimensional model. Body composition should be considered alongside body weight, and strength and physical function should be assessed whenever feasible, particularly in individuals at greater risk of muscle-related decline [5,7,8]. Measures such as appendicular lean mass, handgrip strength, chair-stand performance, gait speed, and related indicators of functional capacity can provide information that the scale cannot [7,8]. In that framework, treatment success is defined not simply by how many kilograms are lost, but by whether fat mass is reduced while muscle-related reserve and metabolic capacity are preserved.

Overall, body weight remains useful, but it is no longer sufficient as the principal endpoint of obesity treatment [5]. In the GLP-1RA era, therapeutic success must be interpreted through the quality of the tissue response, not only through the magnitude of weight change. That shift sets up the next section, which examines more directly the physiological and clinical significance of lean mass, muscle quality, and functional risk during pharmacological weight loss.

## 4. Lean Mass, Muscle Quality and Functional Risk During Pharmacological Weight Loss

Once total body weight is no longer treated as a sufficient descriptor of treatment success, the next question becomes more demanding and more clinically relevant: What is the meaning of the non-fat component that is lost during pharmacological weight reduction? This question has become especially important in the GLP-1RA era because these therapies can induce substantial changes in body mass, yet the scale alone cannot tell us whether the non-adipose component of that change reflects expected adaptation, biologically meaningful skeletal muscle loss, or a more complex remodeling of body composition with uncertain functional consequences [2,3,5]. As discussed in the previous section, some decline in fat-free mass is expected during intentional weight loss [5,6]. The real issue is not whether that happens, but what it means in a given patient, how it should be interpreted, and when it becomes clinically important.

That distinction matters because the biological significance of lean tissue change is not fixed. Heymsfield et al. challenged the widely repeated assumption that weight loss consists of a relatively stable proportion of fat-free mass and showed instead that the composition of weight loss varies according to baseline adiposity, sex, age, and the characteristics of the intervention [13]. In practical terms, the same numerical reduction in non-fat compartments may have very different meanings depending on whether it occurs in a younger patient with substantial adipose reserve and preserved function or in an older adult with sarcopenic obesity, low strength, and limited physiological reserve [6,7,8,13]. Linge et al. brought this issue into the current pharmacotherapy landscape by arguing that muscle-related changes during GLP-1-based treatment should not be classified reflexively as either benign or harmful, but interpreted as potentially adaptive or maladaptive depending on context [14]. The problem is not simply that lean tissue changes occur, but that the meaning of those changes depends on tissue composition, functional reserve, and phenotype-specific vulnerability.

### 4.1. Lean Mass Loss During Pharmacological Weight Reduction: Expected Adaptation or Clinical Concern?

Several consistent patterns emerge when the published evidence is examined comparatively rather than as a sequence of single-study summaries. The proportion of weight loss accounted for by fat-free mass is broadly comparable across selective GLP-1RAs and dual-incretin agonists, typically in the range of 25–40%, yet the absolute amount of lean tissue lost is greater with tirzepatide because the total weight loss is itself larger—a distinction of immediate clinical relevance for individuals whose baseline lean mass already falls in the lower normative range. The functional cost of an equivalent relative decrement in lean mass is also unevenly distributed: in older adults and in patients with sarcopenic obesity, pre-existing anabolic resistance and reduced functional reserve translate the same proportional decline into a substantially larger absolute risk of strength and gait-speed loss. The benefit-risk balance shifts again across glycaemic categories. In patients with type 2 diabetes, the glycaemic and cardiometabolic benefits dominate the overall treatment goal, whereas in normoglycaemic individuals with class I obesity, the preservation of lean mass and physical function carries a proportionally larger share of that goal. Taken together, these observations argue for a phenotype-aware prescription strategy rather than uniform GLP-1RA therapy across clinically heterogeneous populations.

The disproportionate loss of lean mass during pharmacologically induced energy restriction can be traced to a coordinated shift in the canonical pathways that regulate muscle protein turnover. Sustained energy deficit and reduced amino-acid availability blunt the mechanistic target of rapamycin complex 1 (mTORC1) and its downstream effectors p70S6K1 and 4E-BP1, attenuating mRNA translation and the post-prandial rise in muscle protein synthesis. In parallel, the rise in the AMP:ATP ratio activates AMP-activated protein kinase (AMPK), which phosphorylates TSC2 and Raptor to further restrain mTORC1 and which suppresses RNA Polymerase I-driven ribosomal RNA synthesis, narrowing translational capacity over time. Catabolic pathways move in the opposite direction. Energy restriction up-regulates FoxO-dependent transcription of the muscle-specific E3 ubiquitin ligases MuRF1/TRIM63 and Atrogin-1/MAFbx, which target sarcomeric proteins for ubiquitin–proteasome-mediated degradation, and accelerates both macroautophagy and chaperone-mediated autophagy. The net effect is a shift in protein balance toward catabolism. In GLP-1RA-treated patients, the central appetite-suppressive mechanism reduces both total energy and total protein intake, amplifying the AMPK/FoxO axis and producing a state of anabolic resistance—a blunted synthetic response to nutrient and contractile stimuli that is conceptually similar to that observed in ageing skeletal muscle. The countermeasures discussed below—precision protein nutrition and resistance training—are therefore best understood as targeted attempts to reopen the mTORC1/Pol I anabolic window while limiting AMPK/FoxO-driven catabolism.

A certain decline in fat-free mass during negative energy balance is biologically expected and should not, in itself, be treated as evidence of pathological muscle wasting [5,6,13]. Cava et al. emphasized that preserving healthy muscle is an important goal of weight-loss therapy, but also noted that reductions in body mass inevitably alter fat-free compartments because the body is adapting to lower energy intake and, in many cases, lower habitual mechanical loading [6]. Heymsfield et al. made a similar point from a body-composition perspective, showing that the proportion of fat-free mass lost during weight reduction is not constant but depends partly on baseline body composition and the type of intervention being used [13]. Patients with greater initial adiposity may lose a larger absolute amount of fat while preserving a greater proportion of fat-free mass, whereas patients with lower reserve or greater anabolic vulnerability may show a less favorable partitioning of tissue loss [6,13]. This makes the clinical question more precise. The issue is not simply whether lean mass declines, but whether the pattern of tissue change remains proportionate and biologically acceptable in the context of treatment.

This issue has become more visible with modern anti-obesity pharmacotherapy because the magnitude of weight loss is now large enough to make tissue partitioning clinically relevant [2,3]. As summarized by Tinsley and Heymsfield, available body-composition data with GLP-1-based therapies indicate that most of the weight lost is fat mass, but that a measurable fraction also comes from fat-free mass [5]. This observation is important because it corrects two equally reductive interpretations. First, it argues against the notion that GLP-1RA-induced weight loss is simply “pure fat loss.” Second, it also argues against the idea that these therapies intrinsically cause major skeletal muscle depletion. The more accurate interpretation is more nuanced: adiposity is reduced preferentially, but not exclusively, and the non-fat component of that response must be interpreted in relation to the patient’s physiology, reserve, and function [5,6,14].

The trial literature illustrates this point well. In the SUSTAIN 8 body-composition substudy, McCrimmon et al. compared once-weekly semaglutide with once-daily canagliflozin in adults with type 2 diabetes and showed that semaglutide improved body composition primarily through greater reductions in total and visceral fat mass, while lean mass also declined to a lesser extent [15]. What makes this clinically relevant is that it shows pharmacological weight loss is not adipose-selective in absolute terms, yet the dominant direction of change remains favorable from a body-composition standpoint. A similar pattern was observed with tirzepatide. In the SURMOUNT-1 DXA substudy, Look et al. reported that tirzepatide-induced weight reduction in adults with obesity or overweight was composed predominantly of fat mass loss, with roughly three quarters of total tissue loss attributable to fat mass and about one quarter to lean mass [16]. These data do not resolve every mechanistic question, but they do clarify something important: large pharmacologically induced weight loss can still represent a strongly favorable shift in body composition even when some non-fat tissue is lost. The SURPASS clinical trials provide further complementary evidence. Although dedicated body-composition substudies equivalent to SURMOUNT-1 were not uniformly incorporated across the SURPASS series, available pharmacokinetic and body-composition data from tirzepatide trials confirm a predominantly fat-mass-driven weight-loss response broadly comparable to that seen with selective GLP-1RA therapy [16]. The dual GIP/GLP-1 receptor mechanism raises distinct questions about whether incremental GIP receptor engagement modulates fat-free mass partitioning differently from selective GLP-1RA activity, but current data do not support a definitively different compositional profile. This remains an area requiring dedicated head-to-head body-composition analyses.

Real-world semaglutide studies add useful nuance to this interpretation. Rodríguez Jiménez et al. reported that oral and subcutaneous semaglutide in adults with obesity and type 2 diabetes reduced body weight mainly through fat mass loss, with associated reductions in visceral fat area and preservation of phase angle, suggesting a metabolically favorable pattern of tissue change [17]. In a prospective real-life study, Volpe et al. found that once-weekly semaglutide reduced body weight, total fat mass, and visceral adipose tissue over 26 weeks, whereas lean tissue declined more modestly, and handgrip strength was preserved [18]. Their later study of oral semaglutide showed similarly favorable body-composition remodeling, including reduced fat mass and visceral adiposity with preservation of fat-free mass and skeletal muscle mass, leading to an improved skeletal muscle mass-to-visceral adipose tissue ratio [19]. Uchiyama et al. also reported in Japanese adults with type 2 diabetes that oral semaglutide significantly reduced body fat mass without significantly reducing muscle mass over 24 weeks [20]. Viewed together, these studies support a cautious but balanced interpretation. Lean tissue decline is a real component of pharmacological weight loss, but its magnitude and meaning vary, and it should not be interpreted independently of the broader tissue response.

This is why the distinction between measured lean tissue change and clinically meaningful skeletal muscle compromise is so important. Fat-free mass includes body water, organs, connective tissue, and the fat-free fraction of adipose tissue, while DXA-derived lean soft tissue does not directly quantify contractile muscle [5,21]. A reduction in fat-free mass during weight loss should therefore not be read automatically as evidence of muscle dysfunction. At the same time, it should not be dismissed as irrelevant. Its importance depends on whether the patient is also losing reserve, strength, mobility, or the capacity to tolerate daily physical demands. In other words, lean mass loss during pharmacological treatment is expected to some degree, but it becomes clinically meaningful only when interpreted in relation to tissue quality, physical function, and phenotype-specific vulnerability [5,6,13,14,15,16,18,19,20,21].

One of the most important conceptual advances in this area is the recognition that lean mass alone does not define muscle health. Cawthon noted that body-composition measures are useful for estimating tissue quantity, but they provide only partial information about the physiological capacity of skeletal muscle [21]. Koo developed that point more explicitly by distinguishing three related but non-equivalent dimensions of muscle assessment: quantity, quality, and function [22]. This distinction is particularly relevant in obesity treatment because the same patient may lose some lean tissue while maintaining mobility and force production, whereas another patient may appear to retain lean mass despite poor muscle quality, excess adipose infiltration, or impaired neuromuscular efficiency [21,22]. In other words, skeletal muscle quantity and skeletal muscle competence are connected, but they are not interchangeable.

The concept of muscle quality is central here because it explains why the functional meaning of tissue change cannot be inferred from quantity alone. In the Health, Aging, and Body Composition Study, Goodpaster et al. showed that the age-related decline in muscle strength exceeds the decline in muscle mass, implying that qualitative deterioration in the tissue makes a substantial independent contribution to functional loss [23]. Delmonico et al. later extended this perspective by showing that longitudinal strength decline was associated not only with lower muscle quantity, but also with reduced muscle quality and increasing adipose infiltration within muscle [24]. These studies were not conducted in GLP-1RA-treated populations, but they remain highly informative for the present review because they make one point very clear: the biological value of muscle cannot be inferred from mass alone. A reduction in lean tissue during pharmacological weight loss may therefore be less concerning if muscle quality and performance are preserved, whereas apparently stable lean mass may still conceal clinically relevant deterioration if tissue composition and function worsen.

This distinction becomes especially important in obesity, where altered muscle composition is common. Prado et al. argued that sarcopenic obesity represents a particularly unfavorable biological state because reduced muscle reserve and excess adiposity interact to create both metabolic and functional vulnerability [25]. Batsis and Villareal reinforced this interpretation in older adults, emphasizing that sarcopenic obesity is not merely the coexistence of obesity and sarcopenia, but a clinically distinct phenotype with implications for frailty, mobility, and treatment safety [26]. These frameworks are directly relevant to the interpretation of pharmacological weight loss. They imply that a favorable change in total body weight—or even in total fat mass—may still be incomplete if treatment is occurring in a patient whose muscle quality and functional reserve are already compromised.

Early data in GLP-1RA-treated populations illustrate just how important this distinction may become. In the SLIM LIVER study, Ditzenberger et al. reported that semaglutide treatment in people with human immunodeficiency virus and metabolic dysfunction-associated steatotic liver disease was associated with reduced psoas muscle volume over 24 weeks, yet without a significant decline in chair-rise performance or gait speed [27]. This is instructive because it shows that measurable structural muscle change does not automatically translate into short-term functional impairment. Ahmad et al., in a systematic review and meta-analysis of randomized placebo-controlled trials of novel glucose-lowering therapies, reached a similarly cautious conclusion: GLP-1-based treatments appeared to improve some self-reported physical function outcomes, but direct evidence on objective physical performance remained limited and insufficient for firm conclusions [28]. Together, these data suggest that lean tissue decline and functional decline are related but not synonymous outcomes. That distinction is likely to be critical in future obesity trials.

The practical implication is straightforward. The meaning of lean mass change during pharmacological weight loss cannot be reduced to the simple question of whether tissue quantity falls. It must also include what kind of tissue remains and whether that tissue retains the capacity to support force production, mobility, and metabolic function. This is why current sarcopenia frameworks prioritize strength and physical performance rather than muscle quantity alone [7,21,22]. In the context of obesity treatment, the relevant concern is not merely whether some lean mass is lost, but whether the treatment response preserves the functional potential of the body that remains after weight loss has occurred. An important mechanistic dimension that warrants explicit recognition here is myosteatosis—the infiltration of adipose tissue into skeletal muscle. Vieira et al. recently highlighted that poor muscle quality in obesity, including myosteatosis assessed by imaging or derived metabolic constructs, is both prevalent and clinically consequential, yet remains methodologically underdeveloped: definitions span imaging-based intramuscular fat quantification, muscle-specific force output, and broader functional constructs, with no universally accepted measurement standard [28]. In the context of pharmacological weight loss, this matters because reductions in lean tissue quantity do not reveal whether the compositional quality of retained muscle is improving, stable, or deteriorating. A treatment response that reduces subcutaneous and visceral adiposity without addressing intramuscular fat could leave the metabolic and functional quality of skeletal muscle substantially unchanged—an underappreciated dimension of tissue remodeling that conventional body-composition endpoints cannot capture.

### 4.2. Functional Risk Is Phenotype-Dependent During Pharmacological Weight Loss

Perhaps the most clinically important point is that the functional significance of lean tissue loss is not universal. It depends heavily on who is losing the tissue, under what conditions, and against what baseline level of reserve [6,7,8,25,26]. Prado et al. emphasized that sarcopenic obesity creates a high-risk phenotype because the adverse consequences of reduced muscle reserve are amplified by the metabolic and mechanical burden of excess adiposity [25]. Batsis and Villareal made a related argument in older adults, showing that sarcopenic obesity is associated with impaired mobility, frailty risk, and greater complexity of treatment planning [26]. These concepts matter because they make clear that the same degree of non-fat mass loss can be relatively benign in one patient and clinically important in another. In a younger adult with severe adiposity, preserved strength, and good physical function, modest losses in fat-free mass during successful treatment may simply reflect an acceptable adaptation to lower body size and lower mechanical demand [5,6,13]. In contrast, in an older adult with slow gait speed, low baseline strength, prior inactivity, or reduced anabolic responsiveness, a similar reduction may occur on top of already limited reserve and therefore carry much greater functional significance [6,7,8,25,26]. This is one of the strongest arguments against interpreting pharmacological weight loss through the scale or even through total lean mass alone. A given numerical change in body composition does not have a single universal meaning. It only becomes clinically interpretable when placed within phenotype and context. The mechanisms underlying this vulnerability are biologically plausible. Older age, insulin resistance, systemic inflammation, physical inactivity, and blunted anabolic responsiveness all reduce the efficiency with which skeletal muscle adapts to negative energy balance [6,26,29]. In that context, appetite suppression during GLP-1RA therapy may further reduce dietary adequacy unless intake is actively monitored and supported [9,10]. At the same time, weight reduction can lower habitual mechanical loading, while gastrointestinal symptoms may reduce the willingness or capacity to engage in structured physical activity [9,10]. None of this means that GLP-1RAs intrinsically cause functional decline. The more defensible interpretation is that they may create a physiological setting in which pre-existing vulnerability becomes more consequential if nutrition, exercise, and monitoring are not integrated into care. The ESPEN/EASO consensus operationalizes this clinically by requiring both an adiposity criterion and a muscle dysfunction criterion to be simultaneously present. Specifically, sarcopenic obesity is diagnosed when excess adiposity (BMI ≥ 30 kg/m^2^, or waist circumference above validated sex- and ethnicity-specific thresholds) coexists with low muscle mass (e.g., appendicular skeletal muscle mass index <7.0 kg/m^2^ in men and <5.5 kg/m^2^ in women by DXA, or equivalent BIA-derived cutoffs adjusted for equipment and population) and reduced muscle strength (handgrip <27 kg in men, <16 kg in women) or impaired physical performance (gait speed < 0.8 m/s, or SPPB score ≤ 9). This dual-criterion requirement is clinically important because it makes clear that sarcopenic obesity cannot be identified through adiposity or body weight alone, and that muscle function—not just tissue quantity—is a necessary component of the diagnosis.

The broader weight-loss literature provides a useful translational anchor here. Weinheimer et al. concluded that exercise is an important tool for limiting fat-free mass loss during energy restriction in middle-aged and older adults with overweight or obesity [29]. Deutz et al. likewise argued that adequate protein intake, particularly when combined with exercise, is important for maintaining muscle function under catabolic or energy-restricted conditions [30]. These findings do not belong to the pharmacological domain alone, but they are highly relevant because they show that the functional risk associated with lean tissue decline is modifiable rather than inevitable. A patient losing weight under conditions of adequate dietary support, preserved mechanical loading, and structured monitoring is not in the same biological situation as a patient losing weight under low intake, low activity, and no assessment of function [9,10,11,12,29,30].

Overall, lean mass loss during pharmacological weight reduction is common, but its interpretation requires much more nuance than the current public discussion often allows [5,6,13,14]. Muscle quantity, muscle quality, and physical function are distinct but interrelated domains, and the clinical significance of tissue decline depends heavily on phenotype, measurement method, nutritional context, and mechanical loading [21,22,23,24,25,26,27,28,29,30]. The relevant clinical task is therefore not to prevent any reduction in non-fat mass at all costs, but to identify when pharmacological weight loss is occurring in a way that threatens metabolically and functionally important muscle-related reserve. That distinction leads directly to the next section, where the concept of high-quality weight loss is developed as the framework needed to interpret these tissue-level and functional differences in a clinically coherent way.

Table 1 Key body-composition and muscle-related concepts relevant to the interpretation of weight loss quality. These concepts are frequently used in obesity, nutrition, sarcopenia, and exercise literature, but they are not interchangeable. Clarifying their meaning is essential for interpreting the biological and clinical quality of pharmacologically induced weight loss, particularly in the glucagon-like peptide-1 receptor agonist (GLP-1RA) era.

## 5. High-Quality Weight Loss as a New Clinical and Performance Concept

The previous sections establish a clear problem. Total body weight is too crude an endpoint to describe the biological quality of treatment response [5], and changes in lean tissue cannot be interpreted adequately without considering muscle quality, functional reserve, and phenotype-specific vulnerability [6,7,8,13,14,15,16,17,18,19,20,21,22,23,24,25,26,27,28,29,30]. Once those two premises are accepted, the next question follows almost inevitably: what should successful obesity treatment now be aiming for? In the GLP-1RA era, where substantial non-surgical weight loss is increasingly achievable, the field needs a framework that distinguishes mere reduction in body mass from a response that is genuinely favorable from metabolic, musculoskeletal, and clinical standpoints. This is the rationale for the concept of high-quality weight loss. Rather than defining success simply by kilograms lost, high-quality weight loss should be understood as a pattern of response in which excess adiposity is reduced while metabolically and functionally relevant tissue is preserved, physical capacity is maintained or improved, and the overall treatment trajectory remains nutritionally and behaviorally sustainable [5,6,14].

This is more than a semantic refinement. It reflects a broader change in how obesity is being conceptualized and, by extension, how its treatment should be judged. Tinsley and Heymsfield argued that changes in fat-free mass during GLP-1RA treatment must be interpreted within the broader context of body-composition remodeling rather than read in isolation [5]. Linge et al. extended that logic by proposing that muscle-related changes during incretin-based therapy may be adaptive or maladaptive depending on phenotype, treatment context, and whether function is preserved [14]. Together, these perspectives suggest that obesity treatment should no longer be judged only by whether body mass falls, but by whether the body that remains after treatment is biologically more favorable, more metabolically resilient, and more capable of supporting daily physical function. In that sense, high-quality weight loss is not just a more attractive label for standard weight reduction. It is a more demanding and more clinically honest way of defining what therapeutic success should mean. A fifth dimension—bone health—also demands acknowledgment within this framework, particularly given emerging data from pharmacotherapy trials. Body-composition substudies within the GLP-1RA and GIP/GLP-1RA literature, including DXA assessments in SURMOUNT-1 [16], have documented measurable reductions in total and regional bone mineral content alongside fat and lean mass changes. Whether these reductions represent physiologically adaptive skeletal remodeling in response to reduced mechanical loading, or constitute clinically meaningful losses in bone density—particularly in older patients, postmenopausal women, or those with pre-existing osteopenia—remains unresolved. For a framework defining high-quality weight loss in multidimensional terms, the exclusion of bone health from clinical monitoring and from the definition itself is difficult to justify. High-quality weight loss should therefore encompass not only favorable adiposity reduction and lean tissue preservation, but also the absence of clinically significant bone density loss over the treatment course.

### 5.1. Defining High-Quality Weight Loss

Operationally, high-quality weight loss can be characterised as a composite of measurable outcomes evaluated at 6 and 12 months of treatment. The first and necessary criterion is a clinically meaningful reduction in adiposity: at least a 10% relative decrease in fat mass from baseline, ideally documented by DXA, BIA, or quantitative MRI. Preservation of muscle is captured by the second criterion, which requires the absolute decline in appendicular lean mass index (ALMI) to remain within 5% of baseline, with no patient crossing below sex-specific sarcopenia thresholds. The third criterion concerns objective neuromuscular function: handgrip strength, the five-times sit-to-stand test, and habitual gait speed should be maintained or improved relative to pre-treatment values. The fourth focuses on dietary adequacy: protein intake within the target range, fibre intake of at least 25 g per day, and the absence of micronutrient inadequacy on validated screening. The fifth requires the preservation of skeletal integrity, defined as a change in areal bone mineral density of no more than −2% at the total hip and lumbar spine on DXA. The sixth, patient-centred criterion is the preservation or improvement of health-related quality of life, captured with IWQOL-Lite or an equivalent instrument. A patient who meets the adiposity criterion together with at least four of the remaining five criteria is considered to have achieved high-quality weight loss. This definition is offered as a clinical and research starting point, intended to inform both individual decision-making and trial endpoint selection, and is expected to require empirical refinement as direct outcome evidence in GLP-1RA cohorts accumulates.

At the most basic level, high-quality weight loss requires a favorable redistribution of body composition. The dominant component of treatment-induced weight reduction should come from fat mass—particularly visceral and ectopic depots that drive insulin resistance, inflammation, organ dysfunction, and cardiometabolic risk—rather than from disproportionate erosion of lean tissue [2,3,4,5,6]. Yet a definition based on body composition alone is still not enough. As the previous section made clear, fat-free mass is not identical to skeletal muscle mass, and skeletal muscle mass itself is not a sufficient proxy for muscle quality or physical function [5,21,22]. A patient may therefore show a seemingly favorable reduction in fat mass and still have an incomplete response if the treatment also compromises tissue that is important for strength, mobility, or metabolic reserve. High-quality weight loss must therefore be defined not only by what is lost, but also by what is protected and what is functionally preserved.

This is where the distinction between quantity and quality becomes clinically useful. In patients at higher metabolic or musculoskeletal risk, the preservation of function may be more informative than the preservation of tissue mass alone [7,8,23,24]. Goodpaster et al. showed that the decline in muscle strength with aging exceeds the decline in muscle mass, implying that tissue competence deteriorates more rapidly than size alone would suggest [23]. Delmonico et al. similarly demonstrated that worsening muscle quality and increasing adipose infiltration accompany functional decline over time [24]. These studies were not designed to define obesity-treatment success, but they are directly relevant here because they show why a favorable body-composition change cannot be judged solely by kilograms of lean mass retained. From that perspective, high-quality weight loss is best understood as a pattern of change in which adiposity is reduced while the body retains the capacity to generate force, support movement, and sustain metabolic function [7,8,23,24].

This interpretation becomes even more important in phenotypes such as sarcopenic obesity. Prado et al. argued that the coexistence of excess adiposity and reduced muscle-related reserve creates a particularly unfavorable biological state because metabolic and functional vulnerabilities amplify one another [25]. Batsis and Villareal further showed that, in older adults, sarcopenic obesity is associated with mobility limitation, frailty risk, and a greater likelihood that weight loss may become clinically problematic if functionally important tissue is not protected [26]. These observations clarify why high-quality weight loss cannot be a one-size-fits-all concept. In a younger adult with marked adiposity and preserved reserve, aggressive reduction in disease-driving fat depots may be the dominant objective. In an older patient with lower strength, slower gait speed, or limited reserve, the same amount of total weight loss may be much less desirable if it carries a meaningful functional cost. High-quality weight loss is therefore not a fixed formula. It is a clinically calibrated framework for deciding what constitutes a favorable response in a given phenotype [7,8,25,26].

For that reason, high-quality weight loss should be understood as a multidimensional outcome with at least four interrelated components: adiposity reduction, lean tissue preservation, functional maintenance or improvement, and clinical sustainability [5,6,14]. The first refers to the reduction in total fat mass and, importantly, its pathogenic distribution. The second refers to the preservation of tissue that matters for metabolic and physical resilience. The third refers to outcomes such as strength, mobility, and day-to-day performance. The fourth refers to whether the response is achieved in a way that remains compatible with adequate intake, tolerability, and persistence over time. In that sense, high-quality weight loss is broader than a body-composition endpoint and more informative than total body weight alone. It translates tissue biology into a more clinically usable model of therapeutic success.

High-quality weight loss is, first and foremost, a clinical concept because it changes how responses to treatment should be interpreted in practice. Traditionally, the patient who lost more weight was often assumed to have performed better than the patient who lost less. That assumption becomes increasingly unreliable as obesity treatment becomes both more effective and more heterogeneous [2,3,5]. A patient with marked weight loss may still have a suboptimal outcome if that response is accompanied by poor dietary adequacy, disproportionate loss of functionally relevant lean tissue, increasing frailty risk, or limited improvement in physical capability [7,8,25,26]. Conversely, a patient with more moderate total weight loss may still achieve a highly favorable clinical response if disease-driving adiposity is reduced substantially, metabolic markers improve, and physical function is preserved or enhanced [5,14]. In other words, body weight remains relevant, but it can no longer serve as the sole judge of therapeutic value.

Recent semaglutide data help illustrate why this broader perspective matters. In a pooled analysis of the STEP 1–4 trials, Rubino et al. showed that semaglutide 2.4 mg improved physical functioning and weight-related quality of life more than placebo, with a greater proportion of participants achieving clinically meaningful improvement [31]. This is important because it shifts the discussion away from body mass alone and toward outcomes that are closer to patients’ lived experience. The therapy did not simply make participants lighter; it improved domains that speak more directly to how they function and feel. A similar message emerges from the real-world study by Pantanetti et al., in which adults with type 2 diabetes and obesity treated with once-weekly semaglutide showed improvements not only in body weight and body composition, but also in metabolic profile, treatment satisfaction, and quality of life [32]. Importantly, the authors also noted that lean mass changes deserve attention and that parallel strategies to preserve skeletal muscle and function are clinically relevant [32]. These findings reinforce the same point: clinically meaningful treatment success extends beyond what the scale shows.

A further reason this concept is useful is that it accommodates heterogeneity rather than flattening it. Patients differ in age, disease burden, baseline strength, functional reserve, nutritional vulnerability, and treatment priorities [6,7,8,25,26]. A one-dimensional model based on body weight forces all of that variation into a single number. High-quality weight loss does the opposite. It allows clinicians to calibrate treatment goals according to what the patient most needs to gain and what the patient can least afford to lose. For some patients, reducing visceral adiposity and improving glycemic control may be the dominant clinical aim. For others—particularly those with sarcopenic obesity, low reserve, or early functional limitation—preserving mobility and physical independence may be equally central to success [7,8,25,26]. This is one of the main strengths of the concept: it aligns the definition of success with the biology and clinical priorities of the patient in front of us.

This approach is also consistent with recent attempts to define obesity itself in more meaningful clinical terms. The Lancet Diabetes & Endocrinology Commission argued that clinical obesity should be understood not merely through anthropometric thresholds, but through objective evidence of altered tissue, organ, or whole-body function [33]. That proposal is highly relevant here. If obesity-related illness is increasingly understood through biological and functional consequences rather than body size alone, then it follows that successful obesity treatment should also be judged through those same domains. High-quality weight loss fits that logic far better than any purely scale-based criterion. It links the treatment target to the disease model itself.

### 5.2. Why High-Quality Weight Loss Is Also a Performance Concept

High-quality weight loss is not only a clinical concept; it is also a performance concept. That does not mean it belongs exclusively to athletes or to sports nutrition. Rather, it means that obesity treatment should increasingly be interpreted through the patient’s ability to move, generate force, tolerate physical activity, and maintain independence [7,8,21,22,23,24]. In this context, “performance” refers broadly to human function: walking, rising from a chair, climbing stairs, carrying one’s body mass, and sustaining daily physical tasks without disproportionate effort or symptom burden. A treatment response that produces a lower body mass but leaves the patient weaker, less mobile, or less capable would have limited biological and clinical value, especially in populations already close to functional thresholds [7,8,25,26].

This perspective is well supported by the weight-loss and function literature. Straight et al. studied overweight and obese older women undergoing weight reduction and found that improvement in physical function was more strongly related to improved muscle quality than to body weight reduction itself [33]. That finding is especially informative because it illustrates a point that is easy to miss in obesity treatment: the practical value of weight loss lies not simply in being lighter, but in being more capable after weight loss has occurred. Villareal et al. made a similar point in obese older adults by showing that weight loss combined with exercise improved physical function more than weight loss alone [33,34,35]. What mattered most was not the number of kilograms lost in isolation, but the extent to which the intervention preserved or improved the patient’s capacity to function [28,33].

This performance-oriented view becomes even more relevant in the GLP-1RA era because these therapies alter both body mass and energy intake, often relatively quickly [2,3,9,10]. A lower body mass may improve locomotor efficiency, reduce joint loading, and facilitate movement, all of which are favorable for performance. At the same time, if weight loss is accompanied by inadequate intake or reduced mechanical loading in a vulnerable phenotype, the same process may erode muscle-related reserve [9,10,14]. This is precisely why the concept of high-quality weight loss is useful. It integrates the metabolic benefit of adiposity reduction with the musculoskeletal requirement to preserve the body’s capacity to generate force and sustain movement. In this sense, performance is not an optional embellishment to obesity care. It is part of the biological currency through which treatment benefits should be judged.

Recent obesity literature beyond the narrow pharmacological frame supports the same view. Jakicic et al. argued that physical activity and exercise in obesity treatment should be understood as tools for improving health beyond weight loss, including body composition, physical capacity, metabolic health, and long-term maintenance [35]. The more detailed role of resistance training will be developed later in this review, but the conceptual point belongs here: a successful response to obesity treatment is not simply a smaller body, but a body that is metabolically healthier, physically more capable, and more likely to sustain benefit over time [28,33,35]. That broader interpretation is exactly what the term *high-quality weight loss* is intended to capture.

### 5.3. High-Quality Weight Loss as the Organizing Framework for the Rest of the Review

Taken together, the concept of high-quality weight loss provides the framework needed to move from critique to action. Section 3 and Section 4 showed why body weight is insufficient and why lean tissue change requires more careful interpretation. The present section extends that reasoning by proposing that successful obesity treatment should be defined as a multidimensional response characterized by preferential adiposity reduction, preservation of functionally relevant lean tissue, maintenance or improvement of physical function, and support of long-term metabolic and clinical benefit [5,6,14,31,32,33,34,35]. This is not simply an academic refinement. It changes what clinicians should prioritize, what researchers should measure, and what patients should be taught to expect from treatment.

It also provides the organizational logic for the rest of the manuscript. If high-quality weight loss is the target, then the remaining questions become practical rather than purely conceptual. Which nutritional strategies best protect metabolically and functionally relevant tissue during GLP-1RA-induced energy restriction? How should dietary intake be structured when appetite is suppressed, and tolerability becomes a limiting factor? What role should resistance training play in preserving muscle-related reserve? And which outcomes should be monitored if treatment success is no longer defined by total body weight alone? Those questions lead directly into the next sections. In that sense, high-quality weight loss functions as the bridge between the biological critique of weight-centric obesity care and the translational framework needed for precision nutrition, resistance training, and multidimensional monitoring in contemporary obesity treatment [9,10,11,12,35].

## 6. Precision Protein Nutrition During GLP-1-Induced Energy Restriction

If high-quality weight loss is to function as a meaningful therapeutic target rather than a conceptual ideal, nutrition has to be treated as part of the mechanism of treatment success, not as a secondary layer added after pharmacotherapy has already been prescribed. Within that nutritional framework, protein occupies a central place. During GLP-1RA-induced energy restriction, dietary protein becomes the most direct nutritional tool available to help preserve lean tissue while body mass is falling [9,10,11,30]. This matters because the same pharmacological effects that facilitate weight loss—reduced appetite, earlier satiety, smaller meals, and lower spontaneous intake—can also reduce total protein consumption unless intake is planned deliberately [9,10]. Clinically, this means that telling patients simply to “eat less” is no longer an adequate nutritional strategy. What matters increasingly is how the reduced intake is composed, and protein is one of the main determinants of whether that lower-energy pattern remains compatible with muscle preservation and long-term metabolic resilience [10,11,36].

The existing human literature supports this concern. In the meta-analysis by Kokura et al., which included adults with overweight or obesity undergoing intentional weight loss, higher protein intake attenuated declines in muscle mass, although its effects on muscle strength and physical function were less consistent [11]. This distinction is important. Protein appears more reliable as a strategy to protect lean tissue than as a stand-alone intervention to preserve performance, and that limitation should be acknowledged rather than obscured [11]. At the same time, the findings are directly relevant to GLP-1RA therapy because many patients treated with these agents reduce total food intake substantially and may drift toward suboptimal protein exposure without necessarily realizing it [9,10]. In that setting, precision protein nutrition is not about promoting a generic “high-protein diet.” It is about preventing the combination of energy restriction, reduced meal opportunity, and anabolic vulnerability from turning an otherwise favorable weight-loss response into a more muscle-depleting one [9,10,11].

### 6.1. Total Daily Protein Intake: Why the Recommended Dietary Allowance Is Often Not Enough

The first issue is total daily protein intake. The current Recommended Dietary Allowance of 0.8 g/kg/day was designed as a minimum intake to prevent deficiency in generally healthy adults, not as a target for preserving skeletal muscle during pharmacologically assisted weight loss, obesity treatment, aging, or anabolic stress [30,37]. That distinction becomes particularly important under GLP-1RA-induced energy restriction. When total intake is reduced and meal size becomes smaller, the biological question shifts from avoiding deficiency to preserving tissue and function under conditions that may already favor catabolism [9,10,30]. In that context, the Recommended Dietary Allowance is better understood as a lower boundary than as an optimal target.

This interpretation is consistent with the broader protein literature. Paddon-Jones and Rasmussen argued that older adults likely require more protein than the Recommended Dietary Allowance to preserve skeletal muscle, in part because the relevant clinical issue is not simple nitrogen balance but resistance to age-related and inactivity-related anabolic decline [38]. Deutz et al., in the ESPEN expert recommendations, similarly proposed that older adults generally benefit from higher protein intakes, particularly in the presence of illness, inactivity, or other catabolic pressures [30]. Although these recommendations were not developed specifically for GLP-1RA treatment, their logic is highly applicable here. A patient experiencing marked appetite suppression, reduced meal size, and ongoing energy deficit may face a physiological situation in which protein requirements for preserving lean tissue are effectively higher than what the Recommended Dietary Allowance was ever intended to cover [9,10,30,38].

Recent guidance specific to GLP-1RA therapy supports the same shift in thinking. The joint advisory from the American College of Lifestyle Medicine, the American Society for Nutrition, the Obesity Medicine Association, and The Obesity Society identifies adequate protein intake as a priority during GLP-1RA-assisted weight loss, explicitly linking it to the preservation of muscle mass and healthy aging during reduced energy intake [10]. The modified Delphi consensus on supportive care for GLP-1-based therapies likewise emphasizes that nutritional support during both active weight loss and maintenance should include attention to protein adequacy and lean-mass preservation [9]. These recommendations do not imply that every patient requires the same protein target. Rather, they reinforce a more individualized approach in which age, degree of energy restriction, baseline muscle reserve, obesity phenotype, and exercise exposure all inform what “adequate” protein actually means [9,10]. In practical terms, that usually means the Recommended Dietary Allowance should not be treated as sufficient by default during pharmacologically induced weight loss. In practice, a tiered quantitative framework is more actionable than a single population-level target. A minimum intake of ≥1.2 g/kg body weight per day should be the working threshold for most patients undergoing GLP-1RA-assisted weight loss—sufficient to exceed the Recommended Dietary Allowance, provide a meaningful anabolic stimulus during energy restriction, and prevent inadvertent protein deficiency under suppressed appetite. For older adults, individuals with sarcopenic obesity, those with reduced functional reserve, or patients experiencing marked pharmacologically driven appetite suppression, a target of ≥1.5 g/kg/day is more appropriate given the compounded anabolic resistance and catabolic exposure characteristic of these phenotypes. These thresholds represent clinical working targets, not rigid universal prescriptions: the primary goal is to prevent inadvertent protein inadequacy during pharmacologically reduced intake, with individualization applied according to phenotype, tolerance, and treatment phase.

This point becomes even more relevant when obesity-related anabolic resistance is considered. Nilsson et al. reported that obesity and metabolic disease can blunt the anabolic response to protein supplementation and resistance exercise, suggesting that excess adiposity does not necessarily protect against muscle-related decline simply because body size is larger [39]. On the contrary, some patients with obesity may be less responsive to anabolic stimuli than expected, particularly if metabolic dysfunction, inactivity, or age-related anabolic resistance are also present [40]. This has important implications for GLP-1RA therapy. Reduced intake during pharmacological treatment may occur in a biological environment that is already less responsive to dietary protein, meaning that a merely “adequate” intake may not be enough to preserve lean tissue effectively [30,41]. That does not justify exaggerated protein prescriptions, but it does support a more careful and individualized approach.

At the same time, the literature does not support the simplistic conclusion that more protein is always better. Stein et al., in a real-world multimodal obesity treatment program involving a very-low-calorie diet, found that additional protein supplementation did not preserve lean body mass compared with a comparator regimen that already provided approximately 1.0 g/kg normalized body weight per day [42,43]. That study should be interpreted carefully, given the severity of the energy restriction, the magnitude of weight loss, and the body-composition method used [44]. Still, it is useful because it reminds us that protein does not act independently of context. Its effectiveness depends on the depth of the energy deficit, the phenotype of the patient, the method used to assess body composition, and the presence or absence of mechanical loading through exercise. Precision protein nutrition therefore cannot be reduced to a supplement-first approach. It has to be embedded within the broader logic of the treatment plan.

The tiered protein-intake target proposed here (≈1.2–1.5 g/kg/day, rising to 1.5–1.8 g/kg/day in higher-risk phenotypes) is largely extrapolated from the geriatric, sarcopenic-obesity, and very-low-calorie-diet studies rather than derived from direct, large-scale randomised trials in GLP-1RA-treated cohorts. No adequately powered randomised trial has yet tested this quantitative threshold against ad libitum or lower-protein control diets in patients receiving semaglutide or tirzepatide. The recommendation should therefore be read as a mechanistically informed and clinically reasonable benchmark, to be refined as GLP-1RA-specific protein dose–response trials become available. A further practical consideration is that achieving this absolute intake while energy intake is markedly suppressed by appetite-suppressive therapy is itself a substantial behavioural challenge that often requires structured dietary support.

### 6.2. Protein Quality, Per-Meal Dose, and Distribution: Why Total Grams per Day Are Not the Whole Story

Total daily protein intake is the first priority, but it is not the only relevant variable. During GLP-1RA treatment, patients often eat less often, tolerate smaller meals, and compress more of their intake into fewer eating occasions [9,10]. Under those conditions, protein quality, per-meal dose, and distribution across the day become more important because there are fewer opportunities to provide a meaningful anabolic stimulus [36]. Layman argued that total protein intake remains the strongest nutritional determinant of lean mass preservation, but that quality and distribution become increasingly relevant when overall intake is lower, meal size is reduced, or anabolic responsiveness is impaired [36]. That description fits the GLP-1RA setting unusually well: patients may be eating less, eating less often, and doing so in the context of age, metabolic disease, inactivity, or all three.

The importance of distribution is supported by human data. Mamerow et al. showed in healthy adults that distributing protein more evenly across breakfast, lunch, and dinner stimulated 24 h muscle protein synthesis more effectively than a skewed pattern in which most protein was concentrated in the evening meal [37]. This is particularly relevant for patients receiving GLP-1RA therapy, many of whom begin to tolerate breakfast poorly, eat lightly during the day, and then rely disproportionately on a single larger meal later on. From a practical standpoint, a patient may meet a reasonable daily protein target on paper while still delivering that protein in a pattern that is suboptimal for muscle maintenance [37]. Loenneke et al. added to this by showing that more frequent meals providing roughly 30–45 g of protein were associated with greater leg lean mass and knee extensor strength [45]. In community-dwelling older adults, ten Haaf et al. also found that higher total protein intake and a more distributed pattern of intake were associated with better physical functioning and quality of life, particularly in the context of regular physical activity [42]. These findings are not specific to GLP-1RA-treated populations, but they are highly relevant because they show that the pattern of intake can influence the relationship between total protein and clinically meaningful outcomes.

Protein quality matters for similar reasons. Paddon-Jones and Rasmussen emphasized that preserving muscle does not depend only on total grams per day, but also on access to high-quality protein sources that provide essential amino acids in sufficient quantity [38]. Deane et al. recently reinforced that point, highlighting that anabolic responses to protein feeding are influenced by dose, source, digestibility, amino acid composition, and interaction with physical activity [40]. This has immediate practical relevance in GLP-1RA-treated patients, because when meal size is small, food choices have to do more work. If a patient can only tolerate a relatively small breakfast or a light evening meal, that meal must still carry enough anabolic value to matter. From that perspective, high-quality sources such as dairy proteins, eggs, fish, lean meats, soy, or targeted oral supplements become particularly useful when total intake is constrained [10,30,38,40].

Leucine is often discussed in this context because of its role in stimulating mechanistic target of rapamycin complex 1 signaling and postprandial muscle protein synthesis. However, the evidence requires nuance. Zaromskyte et al., in their systematic review of the leucine trigger hypothesis, concluded that support for the hypothesis is mixed rather than universal, varying according to age, protein source, and feeding context [41]. Wilkinson et al. later showed that postprandial leucine dose was associated with postexercise muscle protein synthesis responses in older adults [46]. Taken together, these data suggest that leucine density is relevant, especially in older or anabolic-resistant individuals, but should not be treated as a stand-alone solution. For patients receiving GLP-1RAs, the practical implication is not that leucine should be pursued in isolation, but that smaller meals should ideally contain enough high-quality protein to generate a meaningful anabolic response despite reduced total food volume [37,43,44,46]. A practical quantitative threshold follows from this evidence: approximately 3 g of leucine per meal—achievable with roughly 25–40 g of high-quality protein depending on the source—is the minimum required for meaningful mTORC1 activation and postprandial muscle protein synthesis in older and anabolic-resistant individuals. In GLP-1RA-treated patients whose meal volumes are substantially compressed, this threshold carries particular clinical relevance: a patient tolerating only 100–150 g of food per sitting may fail to reach it through ordinary mixed eating without deliberate prioritization of leucine-dense protein sources such as dairy, eggs, lean meat, or targeted oral supplements. Practitioners should therefore frame protein guidance not only in terms of daily totals, but in terms of per-meal adequacy against this leucine threshold, particularly in older or sarcopenic patients eating fewer, smaller meals.

### 6.3. Precision Protein Nutrition in Practice: Feasibility, Constraints, and Clinical Translation

The real challenge is not defining protein as important in theory; it is making adequate protein intake feasible in the setting of pharmacologically reduced appetite. This is where precision becomes clinically meaningful. Protein nutrition during GLP-1RA therapy should not be reduced to the generic instruction to “eat more protein.” Instead, it should be framed around three practical questions: how much protein can this patient realistically tolerate each day, how should it be distributed across the limited meals they are able to consume, and which foods or supplements provide the highest nutritional value within the patient’s tolerance constraints? [9,10,36]. These are not minor implementation details. They are the difference between a theoretically appropriate dietary prescription and one the patient can actually follow.

The modified Delphi consensus on supportive care for GLP-1-based therapies is particularly helpful in this regard because it emphasizes that nutritional strategies should be adapted across treatment phases rather than delivered as a one-time instruction [9]. During dose escalation, tolerance may be the dominant issue. During active weight loss, lean-tissue preservation may become the main concern. During longer-term maintenance, the challenge may shift toward sustaining intake quality despite reduced appetite and possible behavioral drift [9,10]. This temporal view is important because protein needs to be understood not as a static target, but as a dynamic part of the treatment process. A patient who initially struggles with nausea and meal compression may require a different protein strategy from the same patient several months later, once treatment has stabilized.

This also helps explain why total grams per day, although important, are not the whole story. If clinicians prescribe protein targets without considering tolerability, patients may simply fail to achieve them. If they recommend supplements without assessing whether those supplements displace rather than add to habitual intake, the intended increase in protein may never occur. If they focus exclusively on daily totals while ignoring the fact that most intake is concentrated in one meal, an apparently adequate intake may still provide a suboptimal anabolic signal [37,38,45]. Conversely, a strategically distributed intake of high-quality protein across fewer tolerated meals may be more effective than a higher nominal target that is unrealistic in practice. Precision, in this sense, is not about prescribing the highest number. It is about matching the protein strategy to the patient’s biological risk and actual eating capacity [9,10,36].

The translational implications are particularly important for higher-risk phenotypes. Older adults, patients with sarcopenic obesity, those with low baseline strength, severe energy restriction, or poor habitual protein intake are likely to benefit most from early, structured attention to protein strategy [7,8,25,26,30]. In such patients, protein may need to be prioritized at the start of meals, incorporated into smaller but more nutrient-dense eating occasions, and reviewed repeatedly over time rather than assumed to be adequate [9,10]. Community-based data support the plausibility of this approach. Ten Haaf et al. found that higher total and more evenly distributed protein intake was associated with better physical functioning [42], while Coelho-Júnior et al. reported that relative protein intakes of approximately 1.0–1.2 g/kg/day or higher were associated with better lower-limb physical performance in older adults than lower intakes [43]. Although these studies were not conducted in GLP-1RA-treated populations, they reinforce a clinically relevant principle: protein adequacy matters most in those who are least able to tolerate loss of muscle-related reserve.

Overall, the evidence supports a clear but appropriately nuanced conclusion. During GLP-1RA-induced energy restriction, protein should be regarded as the primary nutritional countermeasure to disproportionate lean tissue loss, but its effectiveness depends on more than total grams alone [9,10,11,30,36,37,38,39,40,41,42,43,44,45,46]. Total intake remains the first priority. However, when appetite is suppressed and eating opportunities are reduced, protein quality, per-meal dose, leucine density, and distribution across the day all become more important. The available evidence also suggests that protein is more consistently linked to preservation of lean mass than to preservation of strength or physical performance on its own [11], which reinforces the broader argument of this review: protein is necessary, but not sufficient, within a high-quality weight-loss model. It works best when embedded in a wider strategy that also includes symptom-aware dietary planning, exercise, and multidimensional monitoring. The next section builds directly on that logic by turning to another determinant of quality of life (QoL) and treatment sustainability in the GLP-1RA era: dietary fiber, satiety, gastrointestinal tolerance, and adherence.

## 7. Dietary Fiber, Satiety, Gastrointestinal Tolerance and Adherence

If protein is the nutritional priority most directly linked to the preservation of lean tissue during GLP-1RA-induced energy restriction, dietary fiber occupies a different but equally important place. Fiber is less central to anabolic support, but highly relevant to the *quality* of the reduced-energy diet itself. In the GLP-1RA era, this matters more than might appear at first glance. These therapies do not simply lower total energy intake; they change how eating feels, how quickly satiety develops, how much food can be tolerated at one time, and, in some patients, how sustainable a structured dietary pattern remains over time [9,10]. In that context, dietary fiber should not be treated as a generic symbol of “healthy eating.” It becomes a functional component of treatment, influencing satiety architecture, bowel regularity, food choice, meal composition, and ultimately adherence [47,48,49,50].

This is especially important because the same mechanisms that make GLP-1RAs effective can also make dietary implementation more fragile. Reduced appetite and earlier satiation can be helpful in lowering energy intake, but they may also compress eating opportunities, narrow tolerated food choices, and lead to an unstructured pattern of eating less rather than eating better [9,10]. At the same time, delayed gastric emptying and gastrointestinal adverse effects—especially nausea, vomiting, bloating, diarrhea, and constipation—can make even nutritionally appropriate advice difficult to execute in practice [51,52,53,54,55,56]. Fiber sits right at the center of that tension. Used well, it may improve dietary quality, satiety regulation, bowel function, and adherence. Used poorly—or introduced too quickly, in the wrong form, or at the wrong phase of treatment—it may worsen fullness, bloating, or upper gastrointestinal discomfort [52,55]. For that reason, fiber in the GLP-1RA setting should be understood as a precision nutritional variable, not as a universally beneficial recommendation applied in the same way to every patient.

### 7.1. Dietary Fiber as a Satiety and Dietary-Quality Tool During Reduced Appetite

A critical evidentiary limitation applies throughout this section: every study cited examining the effects of dietary fiber on appetite, body weight, and adiposity was conducted in populations not receiving GLP-1-based pharmacotherapy. The mechanistic extrapolation from these populations to patients on semaglutide, tirzepatide, or related agents is physiologically plausible—shared pathways of gastric distension, nutrient absorption kinetics, and colonic fermentation remain relevant regardless of concurrent pharmacotherapy—but it constitutes an extrapolation, not established evidence. Direct randomized data on fiber intake in GLP-1RA-treated patients are currently absent from the published literature. Readers and practitioners should interpret the recommendations in this section accordingly.

Dietary fiber has long been associated with lower body weight, reduced dietary energy density, and improved diet quality, but its effects are neither uniform nor interchangeable across fiber types [47,48]. In their systematic review of randomized controlled trials, Wanders et al. concluded that fiber can reduce subjective appetite, energy intake, and body weight, although the size and consistency of these effects vary considerably depending on the type of fiber studied and the design of the intervention [47]. Clark and Slavin reached a similar conclusion, noting that some fibers—notably beta-glucan, lupin kernel fiber, rye bran, whole-grain rye, and broader high-fiber dietary patterns—appeared more consistently related to satiety than others, whereas many acute interventions produced little measurable effect on appetite or subsequent intake [48]. This is important because it reminds us that “fiber” is not a single biological entity. Its physiological behavior depends on viscosity, fermentability, hydration properties, food matrix, and timing of consumption [47,48].

That nuance becomes more clinically relevant during GLP-1RA treatment. In patients whose appetite is already being suppressed pharmacologically, the role of fiber is not simply to push satiety even further. In many cases, that would not even be desirable. Rather, fiber becomes useful as a way of shaping the *quality* and *structure* of reduced intake. High-quality weight loss does not require indiscriminate suppression of hunger at all costs. It requires a lower-energy dietary pattern that remains nutritionally coherent, behaviorally tolerable, and compatible with long-term treatment [9,10]. In that setting, fiber-rich foods can be valuable not because they maximize fullness in a simplistic way, but because they support food patterns centered on minimally processed plant foods, improve meal composition, lower dietary energy density, and help prevent the reduced intake from drifting toward a nutritionally sparse pattern [47,48,49,50,51].

The literature on adiposity outcomes supports this broader view. Jovanovski et al., in a systematic review and meta-analysis of randomized controlled trials, reported that viscous fiber supplementation within calorie-restricted diets improved body weight, body mass index, waist circumference, and body fat in adults with overweight or obesity [49]. Thompson et al. similarly found that isolated soluble fiber supplementation produced modest but significant reductions in body weight, body fat, fasting glucose, and fasting insulin in adults with overweight and obesity [51]. These studies were not conducted in GLP-1RA-treated populations, but they remain directly relevant because they show that fiber can contribute meaningfully to the quality of an energy-restricted diet. The likely mechanisms include reduced dietary energy density, delayed nutrient absorption, greater gastric distension, and, in some cases, colonic fermentation with downstream effects on satiety-related signaling [47,48,49,50,51]. None of these mechanisms should be overstated, but considered collectively, they support the idea that fiber can strengthen the nutritional architecture of a lower-energy dietary pattern.

This is particularly relevant in the GLP-1RA setting because eating less does not automatically mean eating better. Some patients respond to reduced appetite by simply shrinking portions of the same low-fiber, protein-poor, energy-dense foods they were already eating [9,10]. Others stop eating regularly during the day and rely on a narrow set of tolerated foods that may be calorie-light but nutritionally thin. In this context, fiber matters because it helps redirect the conversation. The question is no longer only how to support lower intake, but how to make that lower intake more nutrient-dense, more structured, and more sustainable [9,10,50]. That is why fiber belongs in a review focused on high-quality weight loss. It is not a peripheral healthy-eating theme; it is part of the framework that determines whether pharmacologically reduced intake remains biologically favorable over time.

### 7.2. Gastrointestinal Tolerance During GLP-1RA Therapy: When Fiber Helps and When It Can Aggravate Symptoms

The role of dietary fiber during GLP-1RA treatment cannot be discussed without taking gastrointestinal tolerance seriously. Clinical trial data and post-marketing experience consistently show that gastrointestinal adverse events are the most common side effects associated with this drug class, particularly during dose escalation [52,53,54,55,56]. Gorgojo-Martínez et al., in a multidisciplinary expert consensus, summarized nausea, vomiting, diarrhea, and constipation as the dominant gastrointestinal adverse events and noted that they are typically mild to moderate, transient, and most prominent during the initiation and escalation phases of treatment [52]. Wharton et al. reported a similar pattern with semaglutide 2.4 mg in adults with overweight or obesity, showing that gastrointestinal events were frequent but usually temporary and non-serious [53]. These observations matter because tolerability affects not just comfort, but persistence with treatment and the practical feasibility of any accompanying nutritional strategy [52,53].

This is where fiber becomes clinically more complicated. In patients struggling with constipation, reduced stool bulk, or low intake of plant foods due to markedly reduced food volume, gradual increases in fiber can be helpful, particularly when accompanied by adequate fluid intake [52,55]. In that context, fiber may improve bowel regularity and reduce one of the most frustrating and persistent side effects of GLP-1RA therapy. But that same recommendation cannot simply be generalized across all symptom profiles. In patients with prominent nausea, severe early satiety, bloating, or a pronounced sense of postprandial gastric stasis, a rapid increase in fiber—especially from bulky, highly viscous, or poorly tolerated foods—may worsen discomfort rather than improve it [52,55]. This is exactly why nutritional care during GLP-1RA treatment cannot be formulaic. The usefulness of fiber depends not only on what is recommended, but on *when*, *how*, and *to whom* it is recommended.

Gentinetta et al. addressed this point directly in their dietary recommendations for managing gastrointestinal symptoms in patients treated with GLP-1RAs [55]. Their framework is particularly useful because it moves beyond generic healthy-eating advice and treats symptom burden as something that can be modified through meal structure, food texture, portion size, and nutrient selection. In practical terms, this means that the form in which fiber is consumed matters. Softer, lower-volume, more easily tolerated fiber-containing foods may be appropriate during periods of lingering nausea or pronounced fullness, whereas more bulky or highly fibrous foods may be better introduced once upper gastrointestinal symptoms have stabilized [52,55]. In other words, fiber can support treatment, but only if it is used in a symptom-aware way.

An additional point worth emphasizing is that gastrointestinal adverse events are not the main reason GLP-1RAs work. Lingvay et al. showed that the superior weight loss observed with once-weekly semaglutide relative to other GLP-1RAs was largely independent of gastrointestinal adverse events [54]. Wharton et al. reported a similar message in obesity trials, noting that although weight loss tended to be slightly greater among participants experiencing some gastrointestinal symptoms, those symptoms explained only a small proportion of the overall treatment effect [53]. This matters clinically because it counters a problematic interpretation that still appears in practice: the idea that nausea or food intolerance is somehow a sign that the medication is “working better.” From the perspective of high-quality weight loss, that is the wrong standard. Successful treatment is not defined by discomfort. It is defined by the ability to sustain a lower-energy intake in a way that remains nutritionally adequate, tolerable, and compatible with daily function [9,10,53,54]. An important mechanistic distinction warrants explicit addition here: nausea as a driver of total weight loss and nausea as a driver of dietary quality deterioration are not the same phenomenon and should not be conflated. Even when gastrointestinal symptoms explain only a small proportion of total weight reduction—as the Wharton et al. data confirm—nausea-driven food aversion can substantially reshape what a patient is willing or able to eat. Nutrient-dense foods that are texturally complex, strongly flavored, or higher in fiber—lean meats, legumes, cruciferous vegetables, full-fat dairy—are frequently among the first to become intolerable. The result is a patient losing weight primarily through pharmacological appetite suppression while simultaneously drifting toward a nutritionally sparse, low-protein, low-fiber dietary pattern. This mechanistic distinction matters clinically: it implies that gastrointestinal symptom management is not only about patient comfort or treatment persistence, but about preserving the nutritional architecture that makes pharmacologically induced weight loss biologically high-quality rather than merely numerically impressive.

More recent data in non-diabetic adults with overweight or obesity reinforce the class-wide importance of this issue. Ismaiel et al., in a systematic review and network meta-analysis, confirmed that nausea, vomiting, diarrhea, and constipation are the dominant gastrointestinal adverse events across GLP-1RAs used in this population [56]. Their findings are useful not only because they quantify side-effect burden, but because they reinforce that these symptoms are common enough to shape real-world eating patterns. Once that is acknowledged, dietary counseling—including the management of fiber intake—can no longer be treated as optional. It becomes part of routine therapeutic support rather than something reserved for patients with severe or refractory symptoms [9,10,52,55,56].

### 7.3. Fiber, Adherence, and the Sustainability of High-Quality Weight Loss

One of the main reasons fiber deserves a place in this review is that its role extends beyond satiety or bowel function. Fiber is also closely tied to adherence, and adherence is ultimately what determines whether early treatment success becomes a durable benefit. Miketinas et al., using data from the POUNDS Lost trial, found that dietary fiber intake was one of the strongest predictors of both weight loss and dietary adherence in adults following calorie-restricted diets [50]. Importantly, fiber predicted better outcomes independently of total caloric intake and macronutrient composition, suggesting that its value was not simply that it acted as a marker of eating less or eating “better” in a vague sense [50]. This finding becomes particularly relevant in the GLP-1RA era, where treatment efficacy is still dependent on whether the patient can remain engaged with the dietary pattern over time.

This is where fiber helps distinguish between short-term appetite suppression and durable nutritional structure. During GLP-1RA therapy, many patients report that they are less hungry, tolerate smaller meals, and no longer feel able to eat in their previous pattern [9,10,52,55]. Those changes can facilitate weight loss, but they do not automatically produce a sustainable way of eating. Fiber contributes to sustainability because it supports food patterns that are generally less energy-dense, more micronutrient-rich, and more aligned with long-term cardiometabolic health [47,48,49,50,51]. Just as importantly, it can help make the reduced intake feel more structured and less arbitrary. Instead of simply “eating less and less,” the patient is eating in a way that remains patterned, deliberate, and consistent with both symptom tolerance and nutritional adequacy. That distinction is central to the broader argument of this review. High-quality weight loss depends not only on appetite suppression but also on whether the resulting dietary pattern remains livable.

At the same time, adherence in this context has to be interpreted pragmatically. Fiber is not helpful in the abstract; it is helpful when it is selected in a way the patient can actually tolerate and sustain. For some patients, that may mean emphasizing softer or lower-volume fiber-containing foods earlier in treatment. For others, it may mean a gradual introduction of supplemental fiber once constipation becomes relevant and upper gastrointestinal symptoms have settled [52,55]. It may also mean avoiding common well-intentioned mistakes, such as recommending very large salads, bulky legumes, or high-volume plant meals to patients who are already struggling with delayed gastric emptying and early fullness. In practice, adherence is not improved by giving more advice; it is improved by matching the dietary strategy to the symptom profile and treatment stage of the individual patient.

This has an important translational consequence for the broader manuscript. Fiber should be viewed as a determinant of high-quality weight loss, not because it maximizes weight reduction at any cost, but because it helps sustain a lower-energy dietary pattern that remains nutritionally adequate, gastrointestinally tolerable, and behaviorally durable over time [9,10,50,52,55]. That framing avoids two common errors. The first is treating fiber as a generic health recommendation with no pharmacotherapy-specific nuance. The second is focusing so heavily on symptom management that diet quality becomes secondary. The more useful interpretation is that fiber sits at the intersection of satiety, symptom management, and long-term adherence. For that reason, it is one of the nutritional variables that helps distinguish successful short-term weight loss from high-quality, sustainable weight loss.

Overall, dietary fiber should be considered an important component of nutritional strategy during GLP-1RA-assisted weight loss because it can support satiety architecture, improve dietary quality, promote bowel regularity, and strengthen adherence to a reduced-energy eating pattern [47,48,49,50,51]. At the same time, its role must be individualized according to gastrointestinal symptom profile and treatment phase, since fiber that is beneficial during constipation or stable maintenance may be poorly tolerated during active nausea, bloating, or severe early satiety [52,53,54,55,56]. The clinical value of fiber in the GLP-1RA era therefore lies not in indiscriminately increasing intake, but in using it strategically to support a lower-energy diet that remains tolerable and sustainable. From here, the review moves to the main non-pharmacological countermeasure to disproportionate muscle-related decline during treatment: resistance training.

## 8. Resistance Training as the Main Countermeasure to Disproportionate Lean Mass Loss

If high-quality weight loss is to remain more than a conceptual aspiration, then resistance training has to be treated as a central therapeutic tool rather than as an optional lifestyle accessory. The previous sections have already made two points clear. First, pharmacologically induced weight loss can improve body composition substantially, but its biological quality cannot be judged from body weight alone [5]. Second, nutritional support—particularly adequate protein intake—can help protect lean tissue, but nutrition by itself does not provide the mechanical stimulus required to preserve or improve muscle function during ongoing energy restriction [11,30,36,37,38,39,40,41,42,43,44,45,46]. In that context, resistance training occupies a distinctive position. It is the main non-pharmacological strategy capable of directly targeting the tissue and functional systems most likely to become vulnerable during substantial weight loss: skeletal muscle mass, neuromuscular performance, strength, and physical capacity [12,28,29,30,57,58,59,60].

This matters because the problem is not simply that some non-fat tissue is lost during treatment. The real concern is whether weight loss occurs in a way that progressively erodes force-generating capacity, functional reserve, and the musculoskeletal foundation required for long-term metabolic and physical resilience [6,7,8,14]. Resistance training is uniquely relevant here because, unlike weight loss itself, it does not merely alter the amount of tissue in the body. It changes the biological behavior of that tissue. It provides a repeated anabolic and neuromuscular stimulus during a phase in which the organism is otherwise exposed to reduced energy intake, smaller meals, lower mechanical loading, and, in some patients, lower spontaneous activity [30,35]. In practical terms, this makes resistance training the main behavioral countermeasure to the very aspect of weight loss that most concerns this review: disproportionate decline in functionally relevant lean mass and muscle-related capacity.

The broader literature supports this position consistently. Lopez et al., in a systematic review and meta-analysis across the lifespan, concluded that resistance training improves body composition in people with overweight or obesity and is especially useful for increasing or preserving lean mass while reducing fat mass, even when changes in total body weight are modest [12]. Sardeli et al., focusing on obese older adults undergoing caloric restriction, reported that resistance training attenuated most of the lean body mass loss induced by caloric restriction while allowing similar reductions in total body mass and fat mass to those achieved with caloric restriction alone [57]. More recently, Binmahfoz et al. found in a systematic review and meta-analysis that adding resistance exercise to dietary weight loss preserved fat-free mass, enhanced fat mass reduction, and improved muscular strength without materially altering total body weight loss relative to diet alone [60]. Viewed together, these findings reinforce an important idea: resistance training does not necessarily make weight loss larger, but it often makes weight loss better.

### 8.1. Why Resistance Training Is Mechanistically Different from Weight Loss Alone

The reason aerobic exercise and unstructured increases in daily activity fail to preserve lean mass during severe energy restriction can be derived directly from the very-low-calorie-diet and mechanotransduction literature. Low-load aerobic activity does not generate sufficient sarcomeric mechanical tension to fully activate the load-sensitive arm of mTORC1, which depends on PI3K–Akt and Piezo1-mediated signalling, or the c-Myc/RNA Polymerase I axis that drives ribosomal biogenesis. Under the energy-deficit-induced anabolic resistance described above, in which AMPK and FoxO activity dominate, the mechanical stimulus required to over-ride this catabolic background is substantially higher than under energy-balanced conditions. Resistance training crosses this threshold by imposing high-tension contractions on a defined muscle group, and remains the only well-validated route to reopen the anabolic window during pharmacological energy restriction. Aerobic exercise and increased step counts remain clearly beneficial for cardiorespiratory fitness, glycaemic control, and adiposity reduction, but they cannot mechanistically substitute for resistance training as the principal lean-mass countermeasure.

Translating these physiological arguments into clinical practice requires confronting an uncomfortable real-world fact: adherence to structured resistance-training programmes is notoriously low in patients with severe or sarcopenic obesity. Prospective studies of supervised programmes in these populations typically report six- to twelve-month adherence rates of 30–55%, and free-living programmes generally fare worse. The barriers are well documented and overlap considerably across patients, encompassing musculoskeletal pain, exertional fatigue, cardiovascular and pulmonary comorbidities, mobility limitations, psychological burden, and the practical difficulty of reaching supervised facilities. A clinically useful recommendation must therefore be paired with a realistic implementation plan. Low-threshold entry modalities such as chair-based or aquatic resistance, home-based programmes built around minimal equipment such as resistance bands and body-weight progressions, structured behavioural support, and telehealth supervision are all critical to converting physiological efficacy into clinical effectiveness.

Resistance training matters because it provides something that neither pharmacotherapy nor diet can supply: a direct mechanical and neuromuscular stimulus to skeletal muscle during a period in which the body is otherwise under catabolic pressure [30,61]. During negative energy balance, muscle protein turnover is influenced by reduced energy availability, lower substrate intake, and often reduced habitual loading as body mass declines [6,30]. Resistance exercise counters that environment through repeated mechanical tension, motor unit recruitment, and local metabolic stress, all of which stimulate pathways involved in muscle protein synthesis, structural remodeling, and force production [30,35,61]. Even when hypertrophy is blunted by energy restriction, the training stimulus still provides a biologically meaningful signal that resists passive decline in muscle-related reserve.

This is why resistance training should not be treated as interchangeable with a generic recommendation to “be more active.” Aerobic exercise has major value in obesity treatment and remains important for cardiometabolic health, energy expenditure, and physical conditioning [35]. But when the specific clinical question is how to minimize disproportionate lean mass loss and preserve force-generating capacity during intentional weight reduction, resistance training has the most direct mechanistic relevance. Fragala et al., in the National Strength and Conditioning Association position statement, emphasized that resistance training improves muscle strength, muscle mass, power, and functional performance and should be regarded as a foundational strategy for preserving physical capability, particularly in aging populations [60]. Although that statement was not written specifically for GLP-1RA treatment, its relevance here is obvious: if obesity pharmacotherapy lowers body mass in a way that may also challenge muscle-related reserve, then the most rational behavioral adjunct is the one that directly stimulates muscle retention and neuromuscular adaptation [30,35,61].

Another reason resistance training is so important is that its benefits are not limited to changes in muscle size. Even when increases in lean mass are modest, resistance training can improve force production through neural adaptations, improved motor unit recruitment, better coordination, and greater capacity to generate force relative to body mass [23,24,61]. This is highly relevant in obesity treatment because patients may become lighter while simultaneously becoming more capable in relative terms. That can translate into better chair-rise ability, more efficient walking, improved stair climbing, and less difficulty with everyday physical tasks, even if absolute muscle mass does not increase dramatically during treatment [28,33,57,60]. In other words, resistance training improves not only the amount of tissue preserved, but also the usefulness of that tissue.

This is also why resistance training fits naturally within the performance-oriented definition of high-quality weight loss developed in the previous sections. A favorable treatment response is not just a lower number on the scale. It is a body that is lighter in a biologically advantageous way and still capable of generating force, tolerating movement, and supporting metabolic function [5,7,8,14]. Resistance training is the most direct non-pharmacological intervention for helping that happen. It makes the process of weight loss more selective at the tissue level and more favorable at the functional level. That is why it should be viewed as the main countermeasure to disproportionate lean mass loss, rather than as one optional component among many loosely equivalent lifestyle recommendations [12,30,35,57,60].

### 8.2. Evidence from Weight Loss Trials: Resistance Training Preserves Lean Tissue and Improves Strength Even When Body Weight Falls

The strongest support for resistance training in this context comes from intervention studies in adults with overweight or obesity undergoing caloric restriction or intentional weight loss. Sardeli et al., in their systematic review and meta-analysis of randomized controlled trials in obese older adults, found that resistance training markedly attenuated lean body mass loss induced by caloric restriction while preserving the expected reductions in total body mass and fat mass [57]. This is a particularly important finding because it shows that resistance training does not undermine the central objective of treatment. It does not prevent weight loss in the sense of blocking the reduction in body mass. Rather, it improves the composition of that response by helping to preserve tissue that would otherwise be more vulnerable. Their analysis also suggested potential improvement in strength relative to lean mass, pointing toward benefits that may extend beyond tissue quantity alone [57].

Randomized trials in older adults support a similar interpretation. Nicklas et al. studied overweight and obese older adults undergoing resistance training with and without caloric restriction and found that resistance training improved strength, physical function, and body composition, while the addition of caloric restriction improved mobility without erasing the functional benefit of training [58]. That is a useful result because it challenges the common assumption that weight loss and function are inevitably in tension with one another. Under the right conditions, body mass can fall while performance improves. From the perspective of this review, that is precisely what high-quality weight loss should aim to achieve [58].

Villareal et al. extended this point in a landmark trial of obese older adults assigned to weight loss plus aerobic exercise, resistance exercise, both, or neither. The combined aerobic-plus-resistance condition produced the greatest improvement in functional status, but the resistance-training component was clearly central to preserving strength and supporting overall physical function. The relevance of this study to the GLP-1 era is not that it tested modern anti-obesity pharmacotherapy directly, but that it helps define what a good response to weight loss should look like when judged through function rather than mass alone. The most favorable outcome was not simply the greatest reduction in weight. It was the pattern of treatment that most effectively improved physical capability while weight was being reduced [62]. That logic aligns closely with the argument advanced throughout this review.

Evidence in adults with overweight or obesity outside strictly geriatric settings points in the same direction. Miller et al. reported in a randomized trial that resistance training combined with dietary intervention reduced body fat while preserving lean mass, independently of resting metabolic rate [59]. This is useful because it broadens the evidence base beyond older adults and suggests that the value of resistance training is not confined to populations already classified as frail or sarcopenic. Rather, it appears relevant whenever intentional weight loss is occurring, and the quality of tissue change matters [59]. Wewege et al., in a meta-analysis of healthy adults, similarly found that resistance training reduced body fat percentage, total fat mass, and visceral fat [63]. Although that analysis was not specific to obesity pharmacotherapy or dieting, it remains conceptually relevant because it suggests that resistance training may contribute directly to favorable changes in adiposity as well as to preservation of lean tissue [63].

The most recent meta-analytic evidence reinforces this interpretation. Binmahfoz et al. included randomized controlled trials comparing diet-induced weight loss with and without resistance exercise in adults with overweight or obesity and found that resistance exercise did not significantly alter total body mass change, but did preserve fat-free mass, enhance fat mass reduction, and improve muscular strength [60]. This is especially relevant because it speaks directly to the core question of this review. Resistance training may not cause patients to lose more weight in total, but it can change *what kind* of weight is lost and *what kind of capacity* is retained during that process. That is exactly what distinguishes high-quality weight loss from weight loss in the abstract [60].

### 8.3. Translating Resistance Training into the GLP-1RA Era

Direct randomized evidence specifically examining structured resistance training as an adjunct to GLP-1RA-based obesity treatment is still limited. Most major pharmacotherapy trials with semaglutide or tirzepatide include lifestyle advice and encouragement to increase physical activity, but they do not typically incorporate detailed, supervised resistance-training interventions capable of answering how much lean tissue loss can be prevented by training or which patients benefit most [2,3,5]. This is an important limitation of the field, but it should not be confused with an absence of rationale. Mechanistically and clinically, the case for resistance training is already strong because the problem it addresses—disproportionate loss of muscle-related reserve during weight reduction—has been defined clearly, and the broader weight-loss literature supports resistance exercise as the most relevant countermeasure [12,28,29,30,57,58,59,60,61].

The strongest GLP-1-adjacent evidence currently comes from the post-diet-induced weight-loss trial by Lundgren et al., in which adults with obesity were randomized to exercise, liraglutide, both, or placebo during the maintenance phase after an initial low-calorie diet [64]. The combination of exercise and liraglutide was superior to either treatment alone for maintaining healthy weight loss and improving cardiometabolic outcomes [64]. A later secondary analysis by Jensen et al. showed that combined exercise and GLP-1-based pharmacotherapy also improved physical function and cardiorespiratory fitness more than pharmacotherapy alone [65]. These studies did not isolate resistance training specifically, as the exercise intervention included both aerobic and muscle-strengthening components, but they still provide an important signal. Pharmacotherapy alone does not necessarily optimize physical function, and structured exercise meaningfully improves the quality of the overall response [64,65]. Given the specific role of resistance training in preserving lean mass and strength in other contexts, these findings strengthen the translational case for making it a core part of GLP-1RA treatment rather than a peripheral recommendation. An important contextual qualification is required here. The Lundgren et al. trial used liraglutide—not semaglutide or tirzepatide—and employed it specifically in a weight-loss maintenance design following an initial intensive low-calorie diet phase, rather than as primary obesity pharmacotherapy. The pharmacological profile, receptor selectivity, dosing schedule, and typical clinical use of liraglutide differ meaningfully from once-weekly semaglutide 2.4 mg or tirzepatide, the agents now most commonly used in practice. These distinctions do not invalidate the translational message—that exercise adds meaningful value to pharmacotherapy in terms of weight-loss quality—but they do limit how directly the magnitude and composition of these benefits can be extrapolated to patients currently receiving current-generation agents. Dedicated combination-intervention trials using semaglutide or tirzepatide with structured resistance training remain among the most urgently needed studies in this field [64,65].

The same argument is reinforced by broader obesity-treatment reviews. Jakicic et al. argued that exercise within obesity care should be understood not simply as a way to increase caloric expenditure, but as a means of improving body composition, muscle function, cardiometabolic health, and long-term maintenance [35]. Lowrie et al., in a systematic review and meta-analysis of very-low-energy diets, likewise showed that adding exercise mitigated fat-free mass loss during rapid diet-induced weight reduction [62]. Although the available studies did not allow them to identify a clear advantage of one exercise modality over another, the overall message was still clear: when the energy deficit is large, exercise helps preserve non-fat tissue [62]. Within that broader category, resistance training remains the most plausible and most evidence-supported candidate for specifically protecting muscle-related reserve [12,57,60,61,62].

This is why the translational argument does not need to wait for perfect pharmacotherapy-specific trial data before becoming clinically relevant. The combination of mechanism, non-pharmacological weight-loss evidence, and emerging GLP-1-plus-exercise data is already sufficient to support a practical conclusion: if the goal is not simply weight reduction but preservation of a metabolically and functionally stronger body, resistance training should be included as a core component of treatment whenever feasible.

### 8.4. Program Design, Feasibility, and Why Pragmatic Resistance Training Matters

The value of resistance training is strongest when it can actually be delivered. That means the discussion cannot stop at saying that resistance training works. It also has to address how training can be implemented in real-world obesity care, where patients vary widely in age, baseline mobility, symptoms, training history, pain, confidence, and access to equipment. Fragala et al. emphasized that successful resistance training in older or clinically vulnerable populations depends less on complexity and more on appropriate exercise selection, progressive overload, and sufficient effort to stimulate adaptation [61]. In broad terms, the evidence supports whole-body training performed at least two to three times per week, with major muscle groups trained through progressive resistance and enough effort to produce a meaningful stimulus [30,61]. The exact format can vary, but the underlying principle remains the same: without progression and adequate effort, training is less likely to preserve lean tissue or improve function meaningfully.

This point becomes particularly relevant in the GLP-1RA setting because patients are heterogeneous in ways that matter for exercise prescription. Some will be younger and physically capable but inexperienced with resistance exercise. Others will be older, deconditioned, or limited by joint pain, balance concerns, or low confidence. Some will have access to supervised gym-based programs; others will need home-based or minimally equipped options. None of that weakens the case for resistance training. It simply means that the prescription has to be adaptable. What matters is not that every patient follows the same program, but that the training stimulus is real enough to protect muscle-related reserve during ongoing weight loss.

This is where pragmatic exercise studies become useful. In a randomized controlled pilot trial, Binmahfoz et al. evaluated a home-based resistance training programme in adults with overweight or obesity undergoing dietary weight loss [66]. The programme did not significantly change body-composition outcomes over 12 weeks, but it did improve grip strength, knee extensor force, and sit-to-stand performance relative to weight loss alone [66]. That is a valuable result because it shows that even when a pragmatic home-based intervention is not intense enough to preserve measurable fat-free mass over a short period, it may still protect function. In the context of this review, that matters greatly. Functional preservation is not a secondary bonus; it is part of what defines treatment quality.

At the same time, the limitations of pragmatic training need to be acknowledged. Home-based or minimally supervised programmes may improve accessibility, but they may also reduce exercise intensity, progression, and adherence if they are not well structured [60,66]. Binmahfoz et al. themselves noted that the absence of body-composition effects in their pilot study may have reflected low training intensity, unsupervised delivery, and the relatively small degree of lean tissue loss over the intervention period [66]. This observation reinforces a broader point relevant to the entire review: high-quality weight loss depends not only on whether an intervention is present, but on the quality of its implementation. A vague recommendation to “do some strength training” is not equivalent to a progressive, well-designed programme capable of preserving muscle-related reserve during pharmacological weight loss.

Overall, resistance training is the main non-pharmacological countermeasure to disproportionate lean mass loss because it directly targets the tissue and functional systems most vulnerable during energy restriction [12,28,29,30,57,58,59,60,61,62,64,65,66]. Unlike pharmacotherapy or diet alone, it can preserve or improve strength, support muscle-related reserve, and alter the biological quality of weight loss even when total body mass continues to fall [57,58,59,60]. Direct randomized evidence specifically combining GLP-1RAs with structured resistance training is still limited, but the broader literature already makes the practical direction difficult to ignore. If the aim is high-quality weight loss rather than weight loss in the abstract, resistance training should be considered a central element of treatment, not an optional afterthought. That leads naturally to the next section, which turns from intervention to assessment and asks a practical question that now becomes unavoidable: what should actually be monitored when treatment success is no longer defined by body weight alone?

## 9. Body Composition, Strength and Function: What Should Actually Be Monitored

Once obesity treatment is reframed around the concept of high-quality weight loss, the logic of monitoring has to change with it. The previous sections have shown why total body weight is an incomplete endpoint, why changes in lean tissue cannot be interpreted in isolation, and why nutrition and resistance training matter precisely because they influence the *quality* of the response rather than simply its magnitude [5,6,7,8,9,10,11,12,13,14,15,16,17,18,19,20,21,22,23,24,25,26,27,28,29,30,31,32,33,34,35,36,37,38,39,40,41,42,43,44,45,46,47,48,49,50,51,52,53,54,55,56,57,58,59,60,61,62,63,64,65,66]. The practical implication is straightforward: the scale can no longer be the only instrument used to judge whether treatment is going well. A patient may lose a large amount of body weight while preserving little functional reserve, whereas another may lose less weight in total but achieve a clearly more favourable reduction in adiposity with preservation of strength and physical performance. Once that possibility is acknowledged, the relevant clinical question changes. It is no longer just how often body weight should be measured, but what should be monitored alongside it if the aim is to understand whether the treatment response is biologically and functionally favorable.

**Figure 2:**
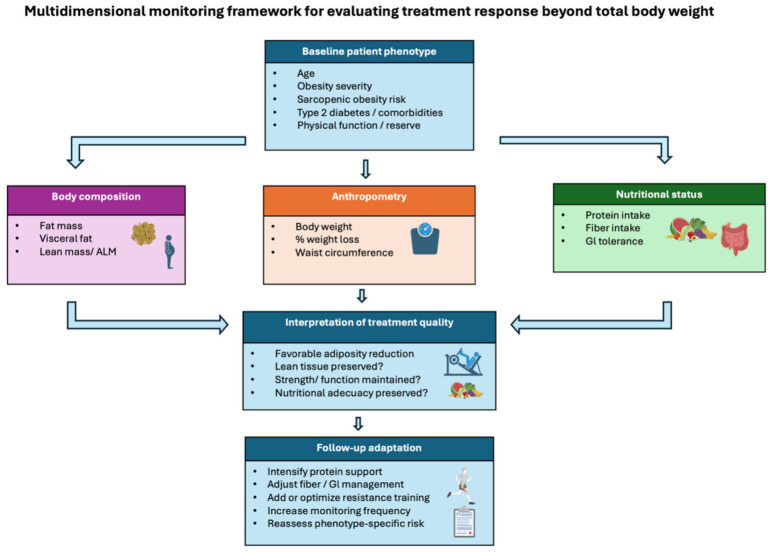
Multidimensional monitoring framework for evaluating treatment response beyond total body weight during GLP-1RA-based obesity therapy. In the glucagon-like peptide-1 receptor agonist (GLP-1RA) era, treatment response should not be interpreted through body weight alone. A multidimensional monitoring model should integrate baseline phenotype, anthropometric outcomes, body composition, nutritional status, and muscle-related assessment in order to distinguish favorable adiposity reduction from biologically or functionally unfavorable tissue loss. This framework emphasizes that treatment quality depends not only on the magnitude of weight loss but also on preservation of lean tissue and muscle-related reserve, maintenance of strength and physical function, nutritional adequacy, and the need for follow-up adaptation according to phenotype-specific risk.

This is not just a methodological refinement. It reflects a deeper shift in what obesity treatment is trying to accomplish. If successful treatment is no longer defined solely by the number on the scale, then the outcomes used to track that success must also move beyond the scale [5,6,7,8,14]. The difficulty, of course, is that no single alternative metric solves the problem. Body composition tells us something about tissue partitioning, but not necessarily about force production or mobility [21,22]. Strength and physical performance are closely linked to daily function, yet they do not fully capture changes in visceral adiposity, appendicular lean mass, or the distribution of non-contractile tissue [7,8,21,22,23,24]. The answer, then, is not to replace body weight with one superior outcome. It is to adopt a multidimensional monitoring framework in which body composition, strength, and function are interpreted together and adapted to the clinical phenotype of the patient [5,7,8,21,22]. Figure 2 summarizes this practical monitoring model for treatment evaluation beyond total body weight.

### 9.1. Body Composition: What Should Be Measured Beyond Total Body Weight

Body composition is the first domain that needs to be considered beyond total body weight because the central argument of this review depends on it. If obesity treatment is to be judged partly by the biological quality of tissue change, then some measure of what tissue is actually being lost becomes essential [5]. In practical terms, the most informative body-composition outcomes are total fat mass, regional adiposity—particularly visceral adiposity when available—and lean tissue compartments that are biologically or functionally relevant, such as appendicular lean mass [5,7,8]. Tinsley and Heymsfield argued that interpretation of treatment response without reference to body composition is increasingly unsatisfactory in the GLP-1RA era, precisely because substantial weight loss may include both favorable adiposity reduction and non-fat tissue change [5]. Their point is not that body composition solves every interpretive problem, but that it prevents the scale from being mistaken for a tissue-level outcome.

The usefulness of body-composition monitoring depends partly on how it is measured. Cawthon noted that DXA is one of the most practical and widely used tools in sarcopenia-related work because it allows estimation of whole-body and regional fat and lean compartments, including appendicular lean mass [21]. At the same time, he emphasized that DXA does not directly measure contractile skeletal muscle or muscle function [21]. Koo made a similar point more broadly, arguing that the assessment of muscle quantity, quality, and function requires conceptual separation because these domains do not map neatly onto one another [22]. This is particularly relevant in obesity treatment. A patient may show a measurable decline in DXA-derived lean tissue without obvious loss of physical performance, while another may show relatively stable lean mass despite clinically meaningful impairment in strength or mobility. Body-composition monitoring therefore improves interpretation, but it does not eliminate the need for clinical judgment.

More advanced imaging methods can refine that interpretation further, although they are often less feasible in routine care. Heymsfield et al. described MRI and CT as the most specific tools for quantifying skeletal muscle and characterizing muscle-adipose relationships, particularly when the goal is to distinguish contractile tissue from adipose infiltration or other non-contractile components [67]. Buckinx et al. also highlighted the methodological pitfalls of muscle-mass assessment and emphasized that different techniques provide overlapping but non-identical information, which complicates the comparison of findings across studies [68]. This point is particularly important in the current pharmacotherapy landscape. A decline in DXA-derived lean tissue is not the same thing as a decline in MRI-defined muscle volume, and neither is equivalent to a decline in actual muscle function [21,22,67,68]. Monitoring body composition therefore improves specificity, but only when its methodological limits are recognized rather than ignored.

Even with those limitations, body-composition monitoring remains indispensable because it can identify patterns of response that total body weight cannot. In many patients, body weight will still be a useful first-line marker of overall response, but it should increasingly be interpreted alongside waist circumference, fat mass change, and—where feasible—appendicular lean mass or other regional measures [5,7,8]. This becomes especially important in phenotypes at greater risk of muscle-related vulnerability, including older adults, patients with sarcopenic obesity, or individuals losing weight rapidly under marked appetite suppression [7,8,25,26]. In those settings, body composition helps answer the clinically meaningful question the scale cannot answer: is treatment preferentially reducing disease-driving adiposity, or is a biologically meaningful proportion of the response being drawn from tissue that should ideally be preserved?

### 9.2. Strength and Physical Performance: Why Function Has to Be Taken Seriously

A clear limitation of the current literature is that GLP-1RA trials have, with few exceptions, reported physical-function outcomes through subjective or self-reported measures—the SF-36 physical component score, the physical-function domain of IWQOL-Lite, and similar instruments—rather than through objective performance-based endpoints. The high-quality-weight-loss framework, however, ultimately rests on whether pharmacological weight loss preserves objective neuromuscular function. Future GLP-1RA trials should therefore systematically incorporate the EWGSOP2-aligned objective performance battery: handgrip dynamometry, the five-times sit-to-stand test, the Short Physical Performance Battery, and habitual gait speed measured over four metres. In higher-risk phenotypes—older adults and patients with sarcopenic obesity—isokinetic knee-extensor strength and stair-climb power can be added where feasible. Until these endpoints are routinely reported, claims of functional preservation during pharmacological weight loss should be interpreted as provisional, and the discussion of function in this manuscript should be read with this evidentiary limitation in mind.

If body composition tells us something about *what* is changing, strength and physical performance tell us much more directly *what that change means* for the patient. This is why contemporary sarcopenia frameworks have moved toward function-first interpretation. The revised EWGSOP2 identifies low muscle strength as the primary feature of probable sarcopenia, with muscle quantity or quality used for confirmation and physical performance used to indicate severity [7]. The ESPEN/EASO consensus on sarcopenic obesity similarly emphasizes that excess adiposity and reduced muscle function may coexist in a clinically important phenotype that cannot be recognized through body mass alone [8]. These frameworks are highly relevant to obesity pharmacotherapy because they imply that successful treatment should not be judged solely by tissue reduction, but by whether the patient is preserving or improving the functions that matter for independence and resilience.

Muscle strength is therefore not a secondary or optional measure. It is one of the most clinically meaningful outcomes that can be monitored while body weight is falling. Handgrip strength is especially useful because it is relatively inexpensive, quick to perform, reproducible when standardized, and clinically interpretable [7,21]. Dodds et al., using pooled data from 12 British studies, provided normative grip-strength values across the life course, showing that this is not merely a research variable but a measure that can be contextualized across age and population groups [69]. Its relevance here lies not only in feasibility, but in the fact that it offers a first practical signal of whether the musculoskeletal consequences of treatment are favorable or concerning. In real-world settings where sophisticated body-composition tools are not always available, a handgrip dynamometer may provide information that is more clinically useful than body weight alone [7,21,69].

At the same time, grip strength should not be treated as sufficient on its own. In many patients with obesity—particularly older adults or those with pre-existing mobility limitation—lower-extremity function and whole-body performance may be more directly relevant to day-to-day capability [7,8,25,26]. Guralnik et al. developed the Short Physical Performance Battery to assess lower-extremity function through balance, chair-rise ability, and gait speed, and showed that poorer scores were associated with disability and future adverse outcomes [70]. Studenski et al. later demonstrated that gait speed is a strong predictor of survival in older adults, reinforcing the clinical meaning of walking performance as more than a descriptive test [71]. These studies were not conducted in GLP-1RA trials, but they remain highly relevant because they make a broader point that applies directly here: physical performance measures are not “soft” outcomes. They are closely linked to trajectories of health, vulnerability, and survival.

This matters in the GLP-1RA era because the relationship between tissue change and function is not always straightforward. Some patients may lose lean tissue while maintaining or even improving relative functional performance, particularly if reduced body mass lowers the mechanical burden of movement and daily mobility becomes easier [14,27,28]. Others may show only modest changes in lean tissue but experience worsening chair-rise performance, gait speed, or overall physical capacity because muscle quality, neuromuscular reserve, or disease burden are limiting the functional response [23,24,27]. This is precisely why function should not be appended as an afterthought once body composition has already been interpreted. It is what tells us whether the tissue response is actually translating into clinical benefit.

A final nuance is that interpretation should consider both absolute and relative performance. During successful obesity treatment, some patients may not become stronger in absolute terms, yet may function more effectively relative to their lower body mass. That is not a trivial improvement; it is often one of the most meaningful clinical gains a patient can experience [28,33,35]. A lighter body with preserved force-generating capacity may climb stairs more easily, stand more efficiently, and move with less effort even if maximal strength values change little. Conversely, a patient who loses weight but does not improve—or who worsens—in function may have a much less favorable response than the scale suggests. This is why performance measures are not simply adjuncts to body-composition monitoring. They are what allow the biological response to be translated into clinical meaning.

### 9.3. A Pragmatic Monitoring Framework: What Should Actually Be Followed in Practice?

The practical implication of all of this is that obesity treatment now requires a layered monitoring strategy rather than a single preferred endpoint. At a minimum, routine follow-up should still include total body weight and, ideally, waist circumference, because both remain useful, accessible markers of overall and central response [1,2,3,4,5]. But if treatment is to be interpreted through the lens of high-quality weight loss, those measures need to be complemented by at least one body-composition assessment strategy and at least one measure of strength or physical performance, especially in patients undergoing marked weight loss or those with baseline muscle-related vulnerability [5,7,8,21,22]. Put simply, body weight still belongs in the monitoring framework, but it can no longer define that framework on its own.

The exact tools used will depend on the clinical setting, resources, and patient phenotype. In specialist clinics or research environments, DXA offers a practical way to quantify changes in fat mass and appendicular lean mass over time, while MRI or CT can provide more specific information when muscle composition or adipose infiltration are central questions [21,22,67,68]. In more resource-constrained settings, bioelectrical impedance analysis may still offer a pragmatic adjunct, provided its limitations are acknowledged, and measurement conditions are kept as consistent as possible [68]. However, even when advanced body-composition technology is not available, clinically meaningful monitoring does not have to collapse back to body weight alone. Strength and function can still be followed with simple tools: handgrip dynamometry, chair-stand testing, gait speed, or a short performance battery [7,8,69,70,71]. This matters from a translational standpoint because it means the move toward higher-quality obesity care does not depend entirely on high-end imaging.

A second practical principle is that monitoring should be phenotype-sensitive. Not every patient requires the same depth of assessment. A younger patient with severe adiposity, preserved function, and no obvious nutritional vulnerability may be followed initially with body weight, waist circumference, and selective body-composition reassessment. By contrast, in an older patient with sarcopenic obesity, low baseline strength, slow gait speed, or rapid treatment-induced weight loss, more deliberate monitoring of appendicular lean mass, handgrip strength, chair-stand performance, and walking function becomes much more important [7,8,25,26]. This is where the concept of high-quality weight loss becomes operational rather than theoretical. The monitoring strategy should reflect not only how much weight the patient is expected to lose, but what the patient is most at risk of losing during that process.

This same logic has important implications for clinical trials. Much of the GLP-1RA literature has appropriately focused on body weight, glycated hemoglobin, and cardiometabolic outcomes [2,3,4]. Those remain important, but they are no longer enough if the field wants to understand the full biological and clinical meaning of treatment response. Future trials should increasingly include body-composition endpoints alongside standardized measures of muscle strength and physical performance [5,14,21,22,27,28]. Without these outcomes, it remains difficult to know when pharmacological weight loss is biologically favorable, when it is largely neutral from a functional standpoint, and when it may become problematic in more vulnerable phenotypes. The solution is not to discard body weight as an endpoint, but to place it within a broader hierarchy of outcomes that reflects both metabolic and musculoskeletal consequences.

Overall, what should be monitored during obesity treatment in the GLP-1RA era is not a single substitute for body weight, but a multidimensional set of outcomes that includes body composition, muscle strength, and physical performance [5,7,8,21,22,67,68,69,70,71]. Body weight remains useful, but it cannot tell us whether adiposity has been reduced in a biologically favorable way or whether muscle-related reserve has been preserved. Body-composition assessment improves tissue-level interpretation, while strength and performance testing improve clinical interpretation. Together, they provide the monitoring framework required if high-quality weight loss is to become a real clinical standard rather than an appealing but under-measured idea. This leads directly to the next section, where the major threads of the review are brought together into a broader precision lifestyle framework integrating pharmacotherapy, nutrition, and exercise.

## 10. Integrating Pharmacotherapy, Nutrition and Exercise into a Precision Lifestyle Framework

By this point, the central argument of the review should be clear. In the GLP-1RA era, successful obesity treatment can no longer be defined simply by the amount of body weight lost [5]. The quality of that response depends on what tissue is lost, what tissue is preserved, how physical function changes, whether dietary adequacy is maintained, and whether the overall therapeutic trajectory remains sustainable over time [6,7,8,9,10,11,12,14]. Seen from that perspective, pharmacotherapy, nutrition, and exercise cannot be treated as separate domains that happen to coexist in obesity care. They need to be understood as interdependent components of the same therapeutic process. A patient does not experience semaglutide or tirzepatide in isolation. The drug acts within a body, within an eating pattern, within a functional phenotype, and within a lifestyle context that will either support or undermine the quality of the response.

This is exactly why a precision lifestyle framework is needed. GLP-1Ras do not merely reduce body weight. They alter appetite, meal size, food tolerance, habitual intake, and, in some patients, the way physical activity and exercise are experienced during treatment [2,3,9,10]. For some individuals, those changes make it easier to build a more favorable metabolic and behavioral pattern. For others, they create new vulnerabilities: low protein intake, poor dietary structure, early fatigue, reduced training exposure, or an excessive reliance on the scale as the main marker of success [9,10,30,36]. The practical implication is straightforward. Pharmacotherapy may be the main driver of weight reduction, but the *quality* of that weight reduction depends heavily on how well nutrition and exercise are integrated around it. In this setting, pharmacotherapy does not replace lifestyle medicine; it raises the importance of delivering lifestyle treatment with greater precision.

A precision lifestyle framework should begin with phenotyping, not with generic advice. That is increasingly consistent with the broader movement in obesity medicine away from a purely anthropometric model and toward a more disease-oriented and function-oriented understanding of obesity [34,72,73]. The Lancet Diabetes & Endocrinology Commission proposed that clinical obesity should be defined not merely by excess body size, but by objective alterations in tissue, organ, or whole-body function [34]. That shift has direct relevance here. If obesity-related illness presents differently across patients, then the treatment strategy should also differ according to what the patient most needs to improve and what the patient can least afford to lose during weight reduction [7,8,25,26,34].

In practical terms, that means baseline assessment should extend beyond body weight and body mass index. A useful starting framework includes at least four domains. The first is adiposity phenotype, including body weight, waist circumference, and, where available, body-composition data that help clarify the burden and distribution of excess fat [5,67,68,69,70,71]. The second is muscle-related vulnerability, including age, sarcopenic obesity risk, baseline strength, gait speed, chair-stand performance, habitual activity, and prior mobility limitation [7,8,21,22,23,24,25,26]. The third is nutritional phenotype, including habitual protein intake, meal structure, diet quality, gastrointestinal symptoms, and practical barriers to maintaining nutrient density under reduced intake [9,10,30,36]. The fourth is behavioral and clinical feasibility, including motivation, comorbidities, pain, access to training, treatment goals, and the likely capacity to sustain changes alongside medication [35,72,73].

This type of phenotyping matters because it changes what counts as a good response. A younger patient with marked visceral adiposity, preserved strength, and no major mobility limitation may tolerate relatively rapid fat loss with less concern about modest lean tissue decline [5,6,13]. By contrast, an older adult with sarcopenic obesity, lower grip strength, slower gait speed, or a history of falls may require earlier and more deliberate attention to muscle-preserving strategies, even if total body weight loss is less dramatic [7,8,25,26]. Likewise, a patient whose main challenge is persistent nausea or severe early satiety may need a different nutritional plan from a patient whose main issue is poor dietary structure and low protein intake [9,10,52,53,54,55,56]. Precision, in this context, does not require sophisticated biomarker-driven classification. It requires thoughtful clinical stratification so that treatment intensity and support are matched to biological and functional risk.

### 10.1. Pharmacotherapy Should Be Co-Prescribed with Nutrition and Exercise, Not Added to a Lifestyle Vacuum

A second principle follows from the first: pharmacotherapy, nutrition, and exercise should be prescribed together from the beginning, not layered onto one another only after problems emerge. This is supported both by clinical guidance and by the logic of the available trial literature. The Obesity Canada clinical practice guideline framed obesity management as a chronic, comprehensive process centered on realistic health outcomes rather than on weight alone [72]. The American Association of Clinical Endocrinologists and American College of Endocrinology similarly presented obesity treatment as long-term disease management that integrates behavioral, nutritional, physical, and pharmacological strategies [73]. More specifically, recent supportive care recommendations for GLP-1RA therapy have emphasized that nutritional assessment, protein adequacy, gastrointestinal symptom management, and physical activity support should be incorporated routinely rather than treated as optional adjuncts [9,10]. The point is not simply that lifestyle still matters. It is that pharmacotherapy works best when it is prescribed within a lifestyle structure rather than into a lifestyle vacuum.

The trial literature supports that view. In STEP 3, Wadden et al. showed that semaglutide 2.4 mg combined with intensive behavioral therapy produced substantial weight loss, reinforcing that effective pharmacological treatment and structured lifestyle support are not competing paradigms [74]. That finding matters because it undermines a false dichotomy that has become increasingly common in real-world discussion: the idea that once highly effective anti-obesity drugs are available, lifestyle support becomes secondary or symbolic. The evidence points in the opposite direction. Pharmacotherapy can strongly influence energy intake and adiposity, but the quality of that response still depends on dietary adequacy, symptom management, and behavioral sustainability [9,10,74].

The same principle is visible in the post-weight-loss maintenance trial by Lundgren et al., where exercise combined with liraglutide was more effective than either treatment alone for maintaining healthy weight loss and improving metabolic outcomes [64]. A secondary analysis by Jensen et al. later showed that combined exercise and GLP-1-based pharmacotherapy improved physical function and cardiorespiratory fitness more than pharmacotherapy alone [65]. These studies did not isolate resistance training specifically, but that is not the key point here. What they show is that pharmacotherapy does not automatically optimize the quality of the response unless it is accompanied by structured behavioral support. Weight loss produced by medication alone is not necessarily the same as weight loss achieved within a supportive nutritional and exercise environment [64,65].

This has implications across treatment phases. During initiation, the main issues may be dose escalation, evolving satiety, and gastrointestinal tolerability, which makes early dietary guidance especially important [9,10,52,55]. During the active weight-loss phase, the main concern may shift toward preserving lean tissue, maintaining protein adequacy, and preventing exercise exposure from falling as intake declines [11,30,36,37,38,39,40,41,42,43,44,45,46,47,48,49,50,51,52,53,54,55,56,57,58,59,60,61,62,63,64,65,66]. During maintenance, the challenge may become sustaining a diet of adequate quality, preventing drift toward low intake without nutrient density, and avoiding the gradual loss of training consistency once the early momentum of treatment slows [9,10,35,64,65]. A precision lifestyle framework helps organize these shifting priorities. It treats obesity care as a dynamic process rather than a one-time prescription.

### 10.2. Integration Only Becomes Real When Monitoring Is Adaptive and Multidisciplinary

A third principle is that integration only becomes clinically meaningful if it is supported by adaptive follow-up. Earlier sections argued that body weight should remain part of assessment, but no longer as the sole determinant of success [5,67,68,69,70,71]. In a precision lifestyle framework, monitoring is therefore not only about confirming that treatment is “working” in the narrow sense of lowering body mass. It is about checking whether the response remains biologically favorable and functionally sustainable over time. That means follow-up should include, whenever feasible, repeated assessment of body composition, dietary adequacy, strength, and physical performance in addition to the scale [5,7,8,21,22,67,68,69,70,71]. Importantly, the depth of that monitoring should vary according to phenotype and risk. Patients with low baseline reserve, rapid weight loss, marked appetite suppression, or significant comorbidity are likely to benefit from closer surveillance of muscle-related and nutritional outcomes than those with lower baseline vulnerability [7,8,25,26].

This kind of care is difficult to deliver well without a multidisciplinary structure. Dietitians are often best placed to identify declining protein intake, poor meal distribution, low fiber tolerance, or progressive aversion to nutrient-dense foods during GLP-1RA treatment [9,10,36,37,38,39,40,41,42,43,44,45,46,47,48,49,50,51,52,53,54,55,56]. Exercise professionals are better equipped to translate the abstract goal of muscle preservation into a realistic resistance-training strategy adapted to age, symptoms, mobility, and adherence constraints [35,57,58,59,60,61,62,63,64,65,66]. Clinicians, in turn, need to integrate those domains with medication titration, comorbidity management, safety, and long-term decisions about treatment continuation or modification [9,10,72,73]. The value of multidisciplinary care is therefore not simply that more professionals are involved. It is that each one addresses a determinant of treatment quality that the others cannot fully manage alone.

An adaptive framework also means that the treatment plan must be revised over time rather than fixed at baseline. A patient who initially tolerates therapy well may later show declining protein intake, progressive training dropout, or worsening strength despite a favorable scale response. Another may begin with marked nausea and poor meal tolerance but become much easier to support nutritionally after dose stabilization [9,10,52,53,54,55,56]. The monitoring framework developed in the previous section exists precisely to identify these trajectories early. In that sense, precision is not about predicting every response in advance. It is about responding intelligently as the patient’s phenotype changes under treatment.

This is one of the strongest practical reasons to move beyond scale-based care. Once treatment is judged only by weight loss, these biologically important divergences can easily be missed. Once treatment is judged by the quality of the response, they become central to decision-making.

### 10.3. A Precision Lifestyle Framework Redefines What Counts as Success

Taken together, the integration of pharmacotherapy, nutrition, and exercise into a precision lifestyle framework changes the meaning of therapeutic success in obesity care. A patient is no longer judged simply by how much body weight has been lost, but by whether that weight loss reflects preferential reduction in disease-driving adiposity, preservation of muscle-related reserve, maintenance of physical function, adequate nutrient intake, and an acceptable degree of tolerability and sustainability over time [5,6,7,8,9,10,11,12,34,35,72,73]. This is a more demanding standard than scale-based obesity care, but it is also a more clinically realistic one. It acknowledges that the same amount of total weight loss can carry very different biological and functional meanings depending on what tissue is lost, what capacity is preserved, and what form of support accompanies the treatment.

This framework also serves as a bridge to the final interpretive stage of the review. Once pharmacotherapy is understood as one component within a broader precision lifestyle model, the central questions are no longer simply how to produce more weight loss, but how to understand the gaps that still prevent this model from being implemented more precisely and more consistently. That is the role of the next section. If high-quality weight loss is the goal, then the most important unanswered questions concern how best to measure treatment quality, how to identify phenotype-specific risk, how to design combination-intervention trials, and how to translate this model effectively into routine care. Those questions define the research agenda that must follow from the conceptual framework developed throughout this review.

## 11. Research Gaps and Future Directions

By this stage, the broad direction of the field is difficult to dispute. GLP-1RA-based therapies can induce substantial and clinically meaningful weight loss, but total body weight alone no longer captures the biological quality of that response [2,3,4,5]. The preceding sections have argued that treatment-induced changes in lean tissue need to be interpreted in relation to muscle quality, strength, physical performance, phenotype-specific vulnerability, dietary adequacy, and exercise exposure [6,7,8,9,10,11,12,13,14,15,16,17,18,19,20,21,22,23,24,25,26,27,28,29,30,31,32,33,34,35,36,37,38,39,40,41,42,43,44,45,46,47,48,49,50,51,52,53,54,55,56,57,58,59,60,61,62,63,64,65,66,67,68,69,70,71]. They have also argued that high-quality weight loss offers a more clinically useful framework than body weight reduction alone because it better reflects the tissues, functions, and long-term outcomes that actually matter [14,31,32,33,34,35]. The question, then, is no longer whether modern obesity pharmacotherapy works in the narrow sense of reducing body mass. The more important question is what still prevents the field from implementing this broader and more biologically meaningful model of treatment with greater precision.

At present, the main limitations are not primarily pharmacological. They are methodological, mechanistic, and translational. In other words, the field has progressed faster in producing weight loss than in explaining its tissue-level consequences, identifying who is most vulnerable to suboptimal adaptation, and determining how best to integrate medication with nutrition and exercise [14,75,76]. This is why future research needs to move beyond the now familiar demonstration that GLP-1RAs reduce body weight. What is increasingly needed is a more refined body of evidence addressing what kind of weight is being lost, in whom, under what clinical and behavioral conditions, and with what implications for muscle-related reserve, physical function, and long-term health [9,10,14,34,75,76]. From that perspective, the main gaps in the field can be grouped into four interconnected domains: measurement and endpoint selection, phenotype-specific risk stratification, combination-intervention trial design, and implementation in real-world care.

### 11.1. Measurement and Endpoint Selection Remain Major Limitations

One of the most persistent problems in this field is that many of the outcomes used to describe treatment response remain too crude for the questions we are now trying to answer. Much of the current debate around “muscle loss” during GLP-1RA treatment reflects the fact that the literature still relies heavily on terms and outcomes that are biologically imprecise or too loosely interpreted. As discussed earlier, *fat-free mass*, *lean mass*, *lean soft tissue*, and *skeletal muscle mass* are not interchangeable constructs [5,21,22]. Yet in both scientific discussion and public discourse, they are often treated as though they were. That may have been tolerable when treatment effects were smaller and less clinically transformative, but it is no longer sufficient in a therapeutic era where weight loss can be substantial enough to reshape body composition in ways that may or may not be favorable.

This problem has already been recognized in the recent literature. Linge et al. argued that the key issue is not simply whether non-fat tissue declines during GLP-1-based treatment, but whether the observed tissue changes represent adaptive or maladaptive remodeling [14]. Karakasis et al., in a recent systematic review and network meta-analysis, similarly showed that body-composition responses to GLP-1RAs and GLP-1/GIP dual agonists are heterogeneous across studies and that more accurate characterization of lean tissue outcomes is needed [75]. These are not minor methodological concerns. If the field continues to rely on imprecise or inconsistently interpreted body-composition outcomes, it will remain difficult to determine when treatment is producing a biologically favorable pattern of tissue loss and when it may be eroding reserve in clinically vulnerable groups [14,75].

A related challenge is the assessment of muscle quality, which is increasingly recognized as one of the most relevant but least standardized domains in obesity-related muscle health. Vieira et al. [77] recently highlighted that poor muscle quality in obesity is common, clinically important, and still methodologically underdeveloped, with definitions ranging from imaging-based myosteatosis to muscle-specific strength and broader functional or metabolic constructs. This is highly relevant for future obesity pharmacotherapy research because the concept of high-quality weight loss cannot be operationalized well if the field remains uncertain about how muscle quality should be measured and interpreted [21,22,23,24,77]. Better standardization is needed not only for research comparability, but also because the meaning of lean tissue change during weight loss may ultimately depend as much on tissue composition and function as on tissue quantity itself.

Physical function represents another major gap. Although body weight and body composition are increasingly reported in obesity pharmacotherapy trials, direct measures of strength and physical performance remain relatively uncommon or inconsistently included [27,28,31,32,65]. Yet from a clinical standpoint, functional measures may be more important than modest changes in lean mass alone, particularly in patients at greater baseline risk [7,8,69,70,71]. If future studies continue to prioritize body weight and metabolic outcomes while leaving muscle strength, gait speed, chair-stand performance, or similar measures underassessed, the field will continue to struggle with the same unresolved question: Does pharmacologically induced weight loss leave the patient metabolically healthier but functionally unchanged, improved, or more vulnerable? That is no longer a secondary question. It sits very near the center of what successful treatment should mean.

### 11.2. Phenotype-Specific Risk and Mechanism-Based Research Are Still Underdeveloped

A second major gap concerns heterogeneity of response. Current obesity pharmacotherapy trials provide strong information about average weight loss, but much less about who is most likely to benefit without unacceptable muscle-related trade-offs. This matters because the biological meaning of lean tissue loss is highly unlikely to be the same in every patient [6,7,8,25,26]. A younger adult with severe adiposity, preserved strength, and good functional reserve may tolerate some reduction in non-fat mass with little meaningful consequence. An older patient with sarcopenic obesity, slower gait speed, low baseline strength, or multiple comorbidities may not [7,8,25,26]. The absence of consistent phenotype-stratified analysis remains one of the main reasons why discussions of GLP-1RA-associated “muscle loss” often generate more heat than clarity.

Recent reviews have begun to frame this issue more carefully. Linge et al. emphasized that the interpretation of muscle-related changes during GLP-1-based treatment depends heavily on context, particularly on baseline phenotype and functional reserve [14]. Mechanick et al. likewise argued that minimizing muscle loss during incretin-mimetic therapy requires attention not only to protein intake and micronutrient sufficiency, but also to physical activity and resistance training, making clear that patient vulnerability is shaped by more than the direct pharmacological action of the drug [76]. These perspectives are important because they move the discussion away from asking whether a medication is intrinsically “good” or “bad” for muscle and toward asking how the same therapy may interact differently with different biological contexts [14,76].

This is also where mechanistic research remains too limited. There is still relatively little human evidence on how GLP-1RA-induced energy restriction interacts with obesity-related anabolic resistance, age-related anabolic resistance, insulin resistance, chronic low-grade inflammation, physical inactivity, or poor baseline muscle quality [30,39,77]. Those factors are all biologically plausible modifiers of treatment response, yet they are rarely integrated systematically into trial design or longitudinal interpretation. More work is needed on whether treatment alters myosteatosis, mitochondrial function, muscle-specific strength, or neuromuscular performance in ways that help distinguish adaptive remodeling from the early stages of clinically meaningful decline [23,24,77]. Without this level of mechanistic and phenotype-sensitive analysis, the field will continue to describe average tissue change without fully understanding which patients are doing well and which are simply becoming smaller.

Future research should therefore be designed less around a single average treatment response and more around clinically meaningful subgroups. Older adults, patients with sarcopenic obesity, people with type 2 diabetes and poor physical function, and individuals with rapid pharmacologically induced weight loss but reduced exercise exposure are all obvious candidates for more focused investigation [7,8,25,26]. The central issue is not just whether heterogeneity exists, but whether the field is willing to treat that heterogeneity as biologically important rather than as statistical noise around a mean effect.

### 11.3. Combination-Intervention Trials Are Now More Important than Additional Drug-Only Efficacy Trials

A third major gap concerns intervention design. The current evidence base strongly suggests that the biological quality of pharmacologically induced weight loss depends not only on the medication itself, but on how nutrition and exercise are integrated with treatment [9,10,11,12,30,57,58,59,60,61,62,63,64,65,66]. Yet direct randomized trials designed specifically to test GLP-1RA therapy in combination with structured resistance training, protein-focused nutritional strategies, or symptom-adapted dietary planning remain surprisingly limited. This is one of the clearest translational weaknesses in the field. Pharmacological efficacy has advanced rapidly, but the science of integrated treatment has not evolved at the same pace.

The need here is no longer simply to ask whether exercise helps or whether protein matters. Earlier sections of this review have already made that broad case. The more useful questions are now more specific and more clinically relevant: how much protein is actually needed under pharmacologically reduced intake, how should it be distributed, which fiber strategies improve tolerance without compromising diet quality, what resistance-training dose is sufficient to preserve muscle-related reserve, and how should these strategies be adapted across initiation, active weight loss, and maintenance? [9,10,11,12,30,36,37,38,39,40,41,42,43,44,45,46,47,48,49,50,51,52,53,54,55,56,57,58,59,60,61,62,63,64,65,66]. These are the kinds of questions that determine whether high-quality weight loss can move from a conceptual ideal to a reproducible clinical model.

Mechanick et al. have already argued that incretin-based obesity therapy should be accompanied by comprehensive nutritional and exercise support [76]. The existing exercise-plus-pharmacotherapy trials point in the same direction, showing that structured exercise improves the quality of the response in ways that pharmacotherapy alone does not fully achieve [64,65]. But these studies still fall short of defining optimal intervention design. Carbone et al., in the broader dietary protein literature, argued that future work should move beyond acute muscle protein synthesis outcomes and better characterize how protein affects clinically relevant endpoints across the lifespan [78]. That recommendation is especially relevant here. Obesity pharmacotherapy research now needs combination trials that connect protein strategy, body composition, strength, physical function, and long-term adherence, rather than continuing to examine these variables in parallel but disconnected ways [78].

Longer-term combination studies are also needed. Many obesity trials now extend to 68 or 72 weeks, and cardiovascular outcome studies such as SELECT have pushed that horizon much further for body weight and cardiometabolic outcomes [4]. Yet much less is known about the longer-term trajectory of body composition, physical function, training participation, and dietary adequacy under prolonged pharmacotherapy. It is entirely plausible that a treatment pattern that appears acceptable at 6 or 12 months may become less favorable over several years if resistance training wanes, dietary quality deteriorates, or muscle-related reserve is progressively eroded in vulnerable patients [14,76]. Future research therefore needs to address maintenance not only as weight-loss maintenance, but as maintenance of tissue quality, function, and nutritional sufficiency.

### 11.4. Implementation Science and Real-World Translation Are Now Central Research Priorities

A fourth major gap lies in implementation. Even if the field increasingly agrees that obesity treatment should move toward a high-quality weight-loss model, there is still too little pragmatic evidence on how to deliver that model consistently in routine care. Supportive care recommendations for GLP-1RA therapy already emphasize protein adequacy, nutritional monitoring, gastrointestinal symptom management, and physical activity support [9,10]. Obesity guidelines similarly support comprehensive chronic disease management rather than medication in isolation [72,73]. But recommending integration is not the same as demonstrating how to implement it effectively across healthcare systems, resource settings, and patient populations. That is now one of the most important translational challenges facing the field.

The unresolved questions are practical, but they are not trivial. Who should receive the most intensive body-composition or functional monitoring? What is the minimum effective resistance-training support that remains feasible in routine care? When is dietitian-led follow-up essential, and when can lower-intensity models work? Which symptom-management strategies actually improve persistence with treatment while preserving diet quality [9,10,35,72,73]? These questions sit squarely within implementation science, and they matter because treatment efficacy in trials does not automatically translate into treatment quality in practice.

Real-world care adds complications that efficacy trials often simplify. A patient may respond well under tightly controlled trial conditions and yet struggle in routine practice with declining protein intake, inconsistent training exposure, poor tolerance, or fragmented follow-up. Conversely, some interventions that look modest in tightly controlled studies may prove valuable if they improve persistence, symptom management, or maintenance in the real world. At present, the field is much stronger on describing what *should* ideally happen than on testing how to make it happen consistently for diverse patients over time. That means future work should not only compare drugs or prescribe nutrients in isolation, but also compare delivery models: supervised versus remote resistance training, standard counseling versus dietitian-led support, stepped gastrointestinal symptom algorithms, and risk-stratified monitoring pathways matched to phenotype and treatment phase [9,10,35,57,58,59,60,61,62,63,64,65,66].

### 11.5. Bone Health Represents an Understudied Dimension of Treatment Response

Pharmacological weight loss can compromise bone health through several converging mechanisms. Energy restriction lowers circulating insulin-like growth factor 1 (IGF-1) and oestradiol, reduces mechanical loading on the skeleton as body mass declines, and in many patients shifts bone remodelling toward net resorption, as reflected in elevated C-terminal telopeptide of type I collagen (CTX) without a parallel rise in the formation marker procollagen type I N-terminal propeptide (P1NP). Adipocyte-derived signalling molecules, including leptin and adiponectin, complicate the picture further, and the incretin axis itself appears to act directly on bone: GLP-1 and GIP receptors are expressed on both osteoblasts and osteoclasts and exert measurable effects on bone turnover. The clinical signal is consistent across trials and meta-analyses, with large pharmacological or surgical weight losses accompanied by detectable falls in areal bone mineral density at the total hip and lumbar spine, and the magnitude of the loss scales with the total mass lost. Three practical implications follow. DXA-based bone-mass surveillance should be a routine component of monitoring in patients with established risk factors for osteoporosis—post-menopausal women, older men, those with a prior fragility fracture, and patients on glucocorticoid therapy. Protein intake within the recommended range, vitamin D repletion to a serum 25-hydroxyvitamin D concentration of at least 75 nmol/L, and calcium intake of 1000–1200 mg per day should be ensured throughout the weight-loss programme. And impact-loading or resistance exercise should be prioritised over purely aerobic activity, because only mechanical-tension-generating exercise provides the osteogenic stimulus required to preserve cortical and trabecular bone during accelerated mass loss.

A fifth research gap is conspicuously absent from current GLP-1RA trial reporting and from most discussions of high-quality weight loss: bone health. Body-composition substudies, including DXA assessments conducted within the SURMOUNT-1 program [16,79], have documented measurable reductions in total and regional bone mineral content during tirzepatide-induced weight loss. Whether these reductions represent physiologically adaptive skeletal remodeling in response to reduced mechanical loading—analogous to the expected lean mass reductions—or constitute clinically meaningful bone density decline, particularly in older patients, postmenopausal women, or individuals with pre-existing osteopenia, has not been systematically evaluated. For a framework that defines high-quality weight loss as a multidimensional clinical standard encompassing adiposity, lean tissue, functional capacity, and bone health, the systematic exclusion of bone endpoints from both routine clinical monitoring and pharmacotherapy trial design is difficult to justify. Future randomized trials should pre-specify longitudinal bone mineral density assessment as a secondary or exploratory outcome, and clinical guidelines should explicitly address skeletal health monitoring in patients at elevated baseline risk.

## 12. Conclusions

The GLP-1RA era has changed the therapeutic landscape of obesity treatment in a meaningful way. For the first time, substantial non-surgical weight loss is now achievable in a large proportion of patients through pharmacological therapy, with effects that extend beyond body mass reduction alone [2,3,4]. That progress is clinically important, but it also makes the limitations of a weight-centered treatment model much harder to overlook. As this review has argued, body weight remains a useful clinical marker, but it is biologically too coarse to capture the quality of the response [5]. It cannot distinguish between a favorable reduction in disease-driving adiposity and potentially unfavorable loss of metabolically and functionally relevant lean tissue, nor can it tell us whether the patient is emerging from treatment stronger, more vulnerable, or simply lighter.

This is why the interpretation of pharmacological weight loss now needs to become more selective. Some reduction in fat-free mass during negative energy balance is expected and should not automatically be pathologized [5,6,13]. However, the significance of that change depends on how it is measured, in whom it occurs, and whether it is accompanied by a decline in strength, physical performance, or broader functional reserve [6,7,8,14,21,22,23,24]. In some patients, the net response may still be clearly favorable because adiposity is reduced predominantly while muscle quality and physical capability are preserved. In others, particularly those with lower reserve, sarcopenic obesity, older age, or nutritional vulnerability, the same pattern of weight loss may carry greater biological and clinical cost [7,8,25,26]. The key issue, then, is not simply whether lean tissue changes during treatment, but whether that change remains proportionate, adaptive, and compatible with long-term health.

Within that framework, the concept of high-quality weight loss becomes more than a useful phrase. It provides a more clinically meaningful way of defining therapeutic success in contemporary obesity care. High-quality weight loss can be understood as a multidimensional response characterized by preferential reduction in excess adiposity, preservation of metabolically and functionally relevant lean tissue, maintenance or improvement of strength and physical function, and support of long-term metabolic and behavioral sustainability [5,6,14,31,32,33,34,35]. This concept matters because it aligns treatment outcomes with what obesity actually disrupts: not only body size, but tissue biology, physical capacity, and whole-body function [7,8,34]. It also offers a framework through which pharmacotherapy, nutrition, exercise, and monitoring can be integrated rather than treated as parallel and only loosely connected domains.

The translational implications of that shift are clear. Precision protein nutrition becomes important because pharmacologically reduced appetite may lower total intake and weaken the anabolic support needed to preserve lean tissue during energy restriction [9,10,11,30,36,37,38,39,40,41,42,43,44,45,46]. Dietary fiber becomes relevant because it helps shape satiety, bowel regularity, gastrointestinal tolerance, dietary quality, and adherence, all of which influence whether reduced intake remains sustainable and nutritionally coherent [47,48,49,50,51,52,53,54,55,56]. Resistance training becomes central because it is the main non-pharmacological countermeasure capable of preserving muscle-related reserve, improving strength, and supporting the functional quality of weight loss even as total body mass declines [12,28,29,30,57,58,59,60,61,62,63,64,65,66]. Monitoring must also evolve accordingly. If treatment success is no longer defined by body weight alone, then body composition, muscle strength, and physical performance need to be brought into routine interpretation whenever feasible [5,7,8,21,22,67,68,69,70,71].

At the same time, the current evidence still demands caution. Pharmacotherapy trials now provide robust evidence for weight-loss efficacy, but considerably less direct evidence on long-term muscle quality, phenotype-specific functional risk, and the optimal integration of medication with nutrition and resistance training [14,27,28,75,76,77,78,79,80]. For that reason, the conclusions of this review should not be overstated. The conceptual and scientific basis for moving beyond a purely weight-centric model of obesity treatment is now well established, but current clinical practice still relies predominantly on body weight as the primary outcome, with limited routine assessment of body composition or functional status. As such, the field has not yet fully resolved how to measure, preserve, and individualize the muscle-related and functional dimensions of that response. Future research should therefore focus less on confirming that GLP-1RAs reduce body weight and more on determining how to preserve muscle-related reserve, how to distinguish adaptive from maladaptive tissue change, and how to implement phenotype-sensitive lifestyle support that improves the biological quality of pharmacological weight loss [9,10,14,34,75,76,77,78].

In summary, successful obesity treatment in the GLP-1RA era should no longer be defined only by the magnitude of weight loss achieved. It should be defined by whether treatment preferentially reduces pathogenic adiposity while preserving the tissues and functions most relevant to long-term metabolic health, physical capability, and clinical resilience [5,6,7,8,14,34]. High-quality weight loss, supported by pharmacotherapy, precision nutrition, resistance training, and multidimensional monitoring, offers a more useful and more defensible framework for interpreting obesity treatment in contemporary practice.

## Figures and Tables

**Table 1:** Key body-composition and muscle-related concepts relevant to the interpretation of weight loss quality.

Concept	Operational Meaning	What It Includes	Main Interpretive Limitation if Used Alone	Relevance to This Review
Body weight	Total body mass measured on the scale	Fat mass, fat-free mass, body water, bone mass, gastrointestinal contents, and other compartments	It does not identify which tissues are being lost or preserved	Useful as a global clinical marker, but insufficient as a standalone endpoint in modern obesity treatment
Fat mass	Total mass of adipose tissue	Subcutaneous, visceral, and ectopic fat depots	It does not capture lean tissue status, muscle-related reserve, or functional capacity	Central to the metabolic benefit of obesity treatment, particularly when visceral and ectopic depots are reduced
Fat-free mass (FFM)	All non-fat components of the body	Skeletal muscle, body water, organs, connective tissue, and mineral-free lean compartments	It is often misinterpreted as equivalent to skeletal muscle mass	Important for interpreting the composition of weight loss, but not a direct measure of muscle tissue or function
Lean soft tissue	Non-bone, non-fat soft tissue estimated by body-composition methods such as dual-energy X-ray absorptiometry (DXA)	Primarily skeletal muscle plus water and organ-related soft tissue	It is not identical to contractile skeletal muscle	Commonly reported in body-composition studies, but should be interpreted cautiously when discussing muscle preservation
Skeletal muscle mass	Mass of skeletal muscle tissue	Contractile muscle tissue is distributed across the body	Estimation depends on the measurement method and model used	More relevant than FFM when discussing muscle preservation during pharmacological weight loss
Appendicular lean mass (ALM)	Lean mass in the arms and legs	Limb-related lean tissue used as a proxy of locomotor muscle reserve	It still does not directly measure muscle quality or function	Clinically useful because it is more closely related to mobility and functional status than whole-body lean mass alone
Muscle quality	Functional and structural competence of muscle relative to its size	Force-generating capacity, tissue composition, adipose infiltration, contractile efficiency, and muscle-specific strength	There is no single universally accepted definition or measurement standard	Critical for understanding why muscle mass alone does not fully explain clinical function or treatment quality
Muscle strength	Capacity of muscle to generate force	Commonly assessed by handgrip strength, knee extensor strength, or other dynamometric tests	It does not directly quantify adiposity or body composition	A clinically relevant indicator of muscle-related reserve and a key component of sarcopenia assessment
Physical performance	Capacity to perform functional tasks	Gait speed, chair-stand performance, Short Physical Performance Battery, timed up-and-go, and related tests	It is influenced by multiple systems beyond muscle alone, including balance, cardiorespiratory fitness, pain, and neurological status	Important for determining whether weight loss is translating into preserved or improved real-world function
High-quality weight loss	A multidimensional pattern of favorable treatment response	Preferential adiposity reduction with preservation of metabolically and functionally relevant tissues and functions	It cannot be captured by body weight alone	Integrative concept that underpins the whole review and reframes how obesity treatment success should be interpreted

## Data Availability

No new data were created or analyzed in this study. Data sharing is not applicable to this article.
